# Integrative and comparative analysis of whole-transcriptome sequencing in circCOL1A1-knockdown and circCOL1A1-overexpressing goat hair follicle stem cells

**DOI:** 10.5713/ab.24.0816

**Published:** 2025-02-27

**Authors:** Jian Wang, Xi Wu, Liuming Zhang, Xiaomei Sun, Wei Sun, Kunzhe Dong, Yongjun Li

**Affiliations:** 1 Key Laboratory of Animal Genetics & Molecular Breeding of Jiangsu Province, College of Animal Science and Technology, Yangzhou University, Yangzhou, China; 2 International Joint Research Laboratory in Universities of Jiangsu Province of China for Domestic Animal Germplasm Resources and Genetic Improvement, Yangzhou University, Yangzhou, China; 3 Immunology Center of Georgia, Medical College of Georgia, Augusta University, Augusta, Georgia, USA

**Keywords:** CircCOL1A1, Differentially Expressed, gHFSCs, Regulatory Axis, Whole-Transcriptome Sequencing

## Abstract

**Objective:**

Hair morphogenesis is tightly related to hair follicle stem cells (HFSCs) proliferation and hair follicle (HF) development. Yangtze River Delta white goats (YRDWG) HFSCs are important for producing superior-quality brush hair (SQBH). Nonetheless, the known regulatory mechanisms are not sufficient to explain YRDWG gHFSCs growth, HF development, and SQBH formation.

**Methods:**

To deeply investigate the interaction networks and mechanisms of circCOL1A1 in the HF development and SQBH formation of YRDWG in detail, we applied whole-transcriptome sequencing and bioinformatics analysis of circCOL1A1-knockdown and circCOL1A1-overexpressing HFSCs from YRDWG. STRING and other databases were used to construct multiple interaction networks. Differentially expressed (DE) genes, DE-miRNAs, and DE-circRNAs were further confirmed via real-time quantitative polymerase chain reaction and Sanger sequencing.

**Results:**

A total of 87 genes, 96 miRNAs, and 135 circRNAs were DE between circCOL1A1-knockdown and circCOL1A1-overexpressing gHFSCs. Functional enrichment, gene ontology annotation and Kyoto encyclopedia of genes and genomes analyses identified marked enrichment of these DE- genes, DE-miRNAs, and DE-circRNAs in the MAPK, PI3K/Akt, and focal adhesion signaling pathways, which are closely associated with gHFSCs growth and HF development. In addition, through interaction network construction, four important regulatory axes were obtained, namely, the chi-circCOL1A1-miR-149-5p-CMTM3-AR, chi-circACTN1- miR-671-5p-MAPK3/COL13A1, chi-circITGA6-miR-18a-5p-FGF1/MAP3K1 and chi-circ COBLL1-miR-30a-5p/miR-128-3p-ITGA6/MAPK14/FGF14 axes.

**Conclusion:**

These novel findings provide a valuable and comprehensive basis for investigating the complex mechanism by which circRNAs participate in and regulate HF development and SQBH formation in YRDWG.

## INTRODUCTION

The skin hair morphogenesis of the animal epidermis is tightly related to hair follicle (HF) structure formation. In goats, skin tissue HF development is critical for cashmere and brush hair production [1,2]. Hair follicle stem cells (HFSCs) are inside and located in the outer root sheaths of the epidermis HF structure and neighbor dermal papilla cells (DPCs), and possesses properties, such as, self-renewal, maintained pluripotency, and multi-directional differentiation potential both *in vivo* and *in vitro*. Owing to these outstanding activities, HFSCs provide physiological and medical value in trichomadesis and developmental biology and can differentiate into skin, HFs, and sebaceous glands, where they play a role in healing skin wounds [3,4]. Yangtze River Delta white goats (YRDWG) hair follicle stem cells (gHFSCs) are important for producing superior-quality brush hair (SQBH). SQBH hair is a widely used product in countries and regions associated with Han culture and Chinese culture, and is recognized the optimal raw material for Chinese calligraphy brush production because of its vivid white color, magnificent luster, and fine elastic characteristics [5]. In a prior investigation, we demonstrated the following: i) chi-miR-149-5p promotes gHFSCs proliferation and differentiation by modulating CMTM3 (CKLF-like MARVEL transmembrane domain-containing family 3)/androgen receptor (AR) axis [6,7]; ii) chi-miR-133a-3p and chi-miR-145-5p simultaneously regulate DUSP6 (dual specificity phosphatase 6) to further regulate gHFSCs proliferation and growth and promote gHFSCs differentiation via modulation of NANOG and SRY-related HMG-box gene 9 (Sox9) expression [8-10]; and iii) circCO1A1 originates from collagen type I alpha 1 chain gene (COL1A1), possesses a stable circular configuration, and can sequester or sponge miR-149-5p and then control the CMTM3/AR network to further suppress gHFSCs proliferation, differentiation, and HF development [11,12]. However, the interaction network relationships among the CMTM3 gene, chi-miR-149-5p, and circCOL1A1 in YRDWG gHFSCs are still unclear, and the regulatory mechanisms of the other genes and noncoding RNAs that influence YRDWG HF development and SQBH formation are not fully understood.

Several genes, such as BMP2, Sox9, β-catenin, Wnt10b, and Krt15, have been reported to be closely associated with HF formation and HFSCs growth and development. Specifically, Bone morphogenetic protein 2 (BMP2), which belongs to the transforming growth factor-β family, can promote mouse hair follicle stem cells (mHFSCs) differentiation via the BMP2/gene encoding phosphate and tension homology deleted on chromosome ten (PTEN) axis [13]. Sox9, an important transcription factor, plays important roles in positively regulating cashmere goat HFSCs proliferation and differentiation, maintaining stem cell pluripotency and features by enhancing the specific marker expressions (Krt15, Krt14, CD34) of gHFSCs [14]. β-catenin is directly regulated by Wnt10b, and is a critical regulatory gene that modulates mHFSCs proliferation and directional differentiation; the Wnt/β-catenin axis is the main “switch” of hair growth [15,16]. These findings suggest that a series of genes are important for HFSCs growth and HF development.

In addition to these vital genes and our reported noncoding RNAs, other noncoding RNAs, including miRNAs, lncRNAs, and circRNAs, are also essential for HFSCs growth and HF formation. i) Specifically, miR-214 and miR-214-3p play major roles in skin tissue self-renewal by targeting enhancer of zeste homolog 2 (ENH2) and the Wnt/β-catenin axis to further regulate human hair follicle stem cells (hHFSCs) proliferation and differentiation [17,18]. miR-205, as a novel regulator of the actomyosin cytoskeleton, could enhance the mechanical properties of mHFSCs in bulges, such as their mechanical forces transduction and resistance to force, to further advance hair regeneration [19]. ii) LncRNA-5322 regulates the PI3K/Akt signaling pathway to stimulate HFSCs proliferation and differentiation by interacting with miR-21 to exert its effects and functions in HFSCs, referred to as the lncRNA-5322-miR-21-PI3K/Akt axis [20], and lncRNA-h19 performs indispensable function in the regulation of HF development by targeting the miR-214-3p/β-catenin axis and promoting DPCs growth in cashmere goats, which can further interact with the HFSCs [21]. iii) circRNA0100 and circRNA1926 can function as miRNA sponges to regulate HFSCs growth and HF development; specifically, they can induce cashmere goat secondary HFSCs to differentiate into the HF lineage via sequestration of the miR-153-3p/KLF transcription factor 5 (KLF5) axis and the miR-148a/b-3p/cyclin-dependent kinase 199 (CDK1) axis, respectively [22,23]. These findings further suggest that noncoding RNAs are important for HFSCs growth and HF development.

However, the known regulatory mechanisms in the areas of HFSCs and HF development research are not sufficient to explain and clarify the gHFSCs growth, HF development, and SQBH formation of YRDWG. Here, to fully elucidate the roles and regulatory networks of circCOL1A1 in gHFSCs growth, HF development, and SQBH formation, we first transfected a circCOL1A1-overexpressing vector and circCOL1A1 siRNA sequence into the gHFSCs to constructed circCOL1A1-overexpressing and circCOL1A1-knockdown gHFSCs, respectively. Next, whole-transcriptome sequencing technology was employed to investigate the genes, miRNAs, and circRNAs expression profiles and functions, in circCOL1A1-overexpressing and circCOL1A1-knockdown gHFSCs. Furthermore, multiple interaction and regulatory networks of the differentially expressed (DE-)genes, DE-miRNAs, and DE-circRNAs were generated and constructed.

## MATERIALS AND METHODS

### gHFSCs culture and preparation

gHFSCs in the YRDWG were harvested and used as in our previous research [6,10,24], that is, isolated then cultured from newborn YRDWG neck skin tissue. We grew gHFSCs in six-well plates (Corning) in DMEM-F12 (Gibco,Waltham, MA, USA), with 20% fetal bovine serum(Gibco) and 2% penicillin-streptomycin (Invitrogen) at 37 °C and 5% CO_2_. All experimental protocols were ethically endorsed by the Yangzhou University (Approval number and ID: 202402003, SYXK [Su] 2021-0026).

### CircCOL1A1 overexpression plasmid construction and RNAi synthesis

CircCOL1A1 overexpression vector was extracted from a preserved plasmid constructed in our previous study [12]. Full-length circCOL1A1 (exon 21–24 of the COL1A1 gene, 873 bp) was cloned and inserted into the pLC5-circRNA overexpression vector (Geneseed, Guangzhou, China) to generate the circCOL1A1 overexpression plasmid. circCOL1A1-SI (circCOL1A1-si3) sequence information was obtained from our previous study [12] and then synthesized and acquired from Gene Pharma. The circCOL1A1-overexpressing vector and circCOL1A1-si3 RNA oligos were independently incorporated into gHFSCs to produce circCOL1A1-overexpressing and circCOL1A1-knockdown gHFSCs.

### RNA extraction and quality control

Total gHFSCs RNA was isolated from individual treatment groups (the negative control of SI [NC-group], circCOL1A1-SI [knockdown; SI-group], the negative control of Over [PL-group], circCOL1A1 overexpression [OV-group]) via several TRIzol kits (Takara, Kusatsu, Japan). The isolated total RNA was analyzed on 1% agarose gels to evaluate degradation and contamination. Subsequently, RNA quality and purity were further assessed via a NanoPhotometer spectrophotometer (Implen, Westlake Village, CA, USA). Finally, the gHFSCs RNA quantification and integrity were separately assessed via a Qubit 2.0 flurometer (Life Technologies, Carlsbad, CA, USA) and a Bioanalyzer 2100 system (Agilent Technologies, Santa Clara, CA, USA). All samples with RNA integrity number (RIN) values ≥9.0 were retained for further library construction and whole-transcriptome sequencing.

### RNA library construction and whole transcriptome sequencing

Among the six gHFSCs RNA samples, we arbitrarily chose four samples, and compiled 9 μg of RNA for sequencing. All the remaining samples were used for subsequent real-time quantitative polymerase chain reaction (RT-qPCR) assays. In total, 3 μg RNA was used for gene sequencing, 3 μg RNA for miRNA sequencing, and 3 μg of RNA for circRNA sequencing. Notably, for miRNA and circRNA sequencing, we eliminated ribosomal RNA via two Ribo-Zero rRNA Removal kits (Epicentre; Moraga, CA, USA), and the resulting residue was purified with ethanol precipitation. For circRNA sequencing, the aforementioned-RNA underwent 3 U of RNase R-based (Epicentre) digestion to eliminate linear RNA. Sixteen RNA sequencing libraries were subsequently prepared via the NEBNext Ultra Directional RNA Library Prep kit for Illumina (NEB) as per the manufacturer’s protocols.

In short, each treated gHFSCs sample underwent fragmentation (~300 nt), prior to first-strand cDNA synthesis via the use of random hexamer primers and M-MuLV Reverse Transcriptase (RNase H). Subsequently, we conducted second-strand cDNA synthesis via DNA polymerase I and RNase H. QIA-quick PCR purification kits (Qiagen, Hilden, Germany) were employed for cDNA fragments purification (the end-repaired fragments and fragments with introduced A bases were especially important for circRNA sequencing), which were then ligated to Illumina sequencing adaptors. Lastly, we size-selected the products (150 to 200 bp paired-end reads) and subjected to PCR amplification via Phusion High-Fidelity DNA polymerase, universal primers, and index (X) primers to generate specific mRNA, miRNA, and circRNA-sequencing libraries on an Illumina HiSeq2500 and HiSeq4000 system (Illumina, San Diego, CA, USA) at Biomics Co., Beijing, China.

### Transcript recognition

Raw data (raw reads) in fastq-file format were initially processed via in-house Perl scripts. Afterward, for genes, clean data were generated by eliminating reads with adaptors using USER enzyme (NEB) and reduced quality from the raw data. In case of miRNAs, clean data were harvested by eliminating the 5′ adaptor and inserted reads while trimming the 3′ adaptor sequence, the poly-A/T/G/C reads, and the reads with lengths <18 and >40 from the raw data. For circRNAs, clean data information was achieved obtained by eliminating reads with adaptors, poly-A/T/G/C reads and reduced-quality reads from the raw data. Moreover, we computed the GC content and the Q20 and Q30 values of the clean data. All subsequent assessments were subsequently performed on high-quality clean data.

For gene transcripts screening, the clean paired reads were aligned and mapped to the goat reference genome, Ensembl website - Capra_hircus Ensembl genome browser 113 (ftp://ftp.ensembl.org/pub/release95/fasta/capra_hircus/dna/Capra_hircus.ARS1.dna.toplevel.fa.gz) with HISAT2 (https://daehwankimlab.github.io/hisat2/) software and obtained 25,144 genes. For miRNA transcripts screening, the clean reads were aligned and mapped to the goat miRNAs database (miRbase, https://www.mirbase.org/) using miRDeep2 (https://github.com/rajewsky-lab/mirdeep2) and obtained 1,046 miRNAs, including 410 previous reported miRNAs and 636 new ones (novel-miRNAs). For circRNA transcripts screening, the clean reads were aligned and mapped to the goat reference genome via the Ensembl website, and the CIRI [25] and BWA-MEM [26] algorithms were employed for screening of junction-site reads to further recognize and validate circRNAs. Thereafter, we obtained 14,785 circRNAs, including 11,402 circRNAs formed via exon back-splicing circularization, 1,265 circRNAs that was the result of intron back-splicing circularization and 2,118 circRNAs that originated from intergenic_region back-splicing circularization.

### Differentially expressed-genes, differentially expressed-miRNAs and differentially expressed-circRNAs

Gene expressions were normalized then computed via fragments per kilobase of transcripts per million (FPKM: cDNA fragments/[mapped fragments × transcript length]). The miRNA and circRNA contents were normalized then computed via transcripts per million (TPM; the read-counts ×1,000,000/mapped-reads). To identify DE-genes, DE-miRNAs, and DE-circRNAs between the NC-groups and SI-groups and between the PL-groups and OV groups, the DE-genes, DE-miRNAs and DE-circRNAs were identified then harvested using the DESeq2 package [27]. DE-genes were defined as a fold change (FC) of ≥0.5 and false discovery rate (FDR) of ≤0.05 were deemed to be DE. DE-miRNAs were defined as FC of ≥1.0 and FDR of ≤ 0.01. DE-circRNAs were defined as FC of ≥1.5 and FDR of ≤0.01.

### Differentially expressed-genes, differentially expressed-miRNAs and differentially expressed-circRNAs functional annotation

The RNAhybrid (*v*2.1.2; https://bibiserv.cebitec.uni-bielefeld.de/rnahybrid/) and miRanda (*v*3.3a; https://www.bioinformatics.com.cn/local_miranda_miRNA_target_prediction_120) websites were employed for the DE-miRNAs target gene identification. The topGO R package was used for Gene Ontology (GO) enrichment assessments to determine the biological process (BP), cellular component (CC), and molecular function (MF) terms with DE-genes, DE-miRNAs target genes and DE-circRNAs source genes. Furthermore, Kyoto encyclopedia of genes and genomes (KEGG) assessment was employed for annotation of the DE-genes, DE-miRNAs target genes and DE-circRNAs source genes mediated functional networks. GO terms and KEGG networks with adjusted p-values ≤0.05 or false discovery rates ≤0.01 were considered to have high enrichment, and the top 20 enriched GO terms of each treated gHFSCs are presented in the Figures 2, 4, and 6.

### Interactive network construction of DE-circRNAs, miRNAs and genes

Using the STRING (*v*12.0; https://cn.string-db.org/), BLAST software (*v*2.14.0) and the circRNA-seq data from our whole-transcriptome sequencing, we constructed a protein interaction network of DE-circRNAs, source genes and proteins in circCOL1A1-knockdown and circCOL1A1-overexpressing gHFSCs with corrected p-value<1×10^−10^. Meanwhile, the circRNA-seq and miRNA-seq information were assessed via the RNAhybrid and miRanda websites, and the top 20 up-regulated and top 20 down-regulated circRNAs in circCOL1A1-knockdown and circCOL1A1-overexpressing gHFSCs were selected to obtain the DE-circRNAs-miRNAs interaction network. Finally, in conjunction with the DE-circRNAs, DE-miRNAs and DE-genes analyses from whole-transcriptome sequencing, we constructed competing endogenous RNAs (ceRNAs) interaction networks of circRNAs-miRNAs- genes in circCOL1A1-knockdown (NC-group versus SI-group) and circCOL1A1-overexpressing gHFSCs (PL-group vs. OV-group).

### Identification and verification of differentially expressed-genes, differentially expressed-miRNAs and differentially expressed-circRNAs

Six gHFSCs RNA samples (four from library construction and two from remaining samples) from each treated group were analyzed to further validate the accuracy of the whole-transcriptome sequencing. gHFSCs RNA was converted to cDNA via a Takara 047A Prime-Script RT Kit with a gDNA eraser. 7 DE-genes, 11 DE-miRNAs and 11 DE-circRNAs were selected in circCOL1A1-knockdown gHFSCs (NC-group vs. SI-group), and 9 DE-genes, 11 DE-miRNAs and 11 DE-circRNAs were chosen in circCOL1A1-overexpressing gHFSCs (PL-group vs. OV-group) to further confirm accuracy of the sequencing data. GAPDH and 18S-rRNA were used for genes, circRNAs and miRNAs quantification and as internal references. RT-qPCR reactions were performed in triplicates on an ABI 7500/7500-Fast Real-Time PCR system (Applied Biosystems) with a Takara 820A TB Green II Master Mix Reagent Kit (Takara). The selected DE-genes, DE-miRNAs, and DE-circRNAs expression profiles were quantified via the 2^−ΔΔCt^ formula [28,29]. The primers sequences for the DE-genes, DE-miRNAs and DE-circRNAs are listed in Supplements 1–3. Furthermore, 4 DE-circRNAs were randomly chosen for assessment, and the splice junction site sequences were confirmed via Sanger sequencing.

### Statistical analysis

Data are presented as the Mean-value of 3 technical repeats±standard error (SEM-value) based on at least four or six same samples in each treatment. The NC-group vs. SI-group and PL-group vs. OV-group assessments were carried out via two-independent-samples/groups T-tests in SPSS v24 (IBM) and Origin64(R) 2022 (Origin Lab) software. A p-values < 0.05 were significant threshold, and p-values <0.01 were exceptionally significant threshold. * p<0.05 and ** p<0.01.

## RESULTS

### Identification of genes, miRNAs and circRNAs in circCOL1A1-treated gHFSCs via whole-transcriptome sequencing

Following organization and filtration the raw reads, we collected 817,820,673 high-quality reads in the sum of the gene-seq and circRNA-seq parts, with a total of 205,014,907 high-quality clean reads in the miRNA-seq part in the circCOL1A1-knockdown and circCOL1A1-overexpressing gHFSCs (Supplements 4, 5). Among them, 443,535,128 reads of gene and circRNA, 103,673,318 reads were from the NC and SI-groups; 384,285,545 reads of gene and circRNA, 101,341,589 reads were from the PL and OV-groups. The bases recognition quality and accuracy (Q30-level) of each gHFSCs sample reached 94.80% for both the gene-seq and the circRNA-seq samples and reached 97.40% for the miRNA-seq samples. Up to 97.00% of the gene clean reads, 97.60% of the circRNA clean reads and 66.48% of the miRNA clean reads underwent mapping to the goat reference genome in circCOL1A1-knockdown and circCOL1A1-overexpressing gHFSCs (Supplements 6–8).

### Characteristics of genes, miRNAs and circRNAs in circCOL1A1-treated gHFSCs

In total, 25,144 genes were identified in all treated gHFSCs samples, and their expression density and distribution were calculated as log10-FPKM values, as shown in Supplements 9A, 9B. All genes Pearson’s correlation coefficients (PCC) and FPKM correlations for all the treated gHFSCs samples are shown in Supplements 9C, 9D. The expression tendencies of 12 genes were obtained via K-means cluster analysis, and genes in the same clusters might play similar roles in gHFSCs (Supplement 9E). A total of 1,046 miRNAs were identified in all treated gHFSCs samples, and their expression density and distribution were calculated as log10-TPM values and are provided in Supplements 10A, 10B. All miRNAs PCC and TPM correlations of all the treated gHFSCs samples are shown in supplements 10C, 10D. The nucleotide bias of 410 known-miRNAs is shown in Supplement 10E, and their lengths were distributed mainly between 20 nt and 23 nt (Supplement 10F). The nucleotide bias of 636 newly identified miRNAs is shown in Supplement 10G, and their lengths were distributed mainly from 18 nt to 25 nt (Supplement 10H). In all, 14,785 circRNAs were recognized in all treated gHFSCs samples. The majority of the circRNAs were exonic circRNAs; 11,402 circRNAs came from protein-coding exons of their source genes; few circRNAs were intronic and intergenic; 1285 and 2118 circRNAs originated from the introns and intergenic-regions of their source genes, respectively (Supplements 11A, 11B). The symmetric distribution and expression profile of these circRNAs in all treated gHFSCs samples were computed as log10-TPM values and are provided in Supplements 11C, 11D. All circRNAs PCC and TPM correlations of all the treated gHFSCs samples are shown in Supplements 11E, 11F. The 16 circRNAs expression tendencies were via the K-means cluster analysis, and circRNAs in the same clusters might have similar regulatory functions in gHFSCs (Supplement 11G).

### Differentially expressed-genes analysis in all treated gHFSCs samples

**Figure 1:**
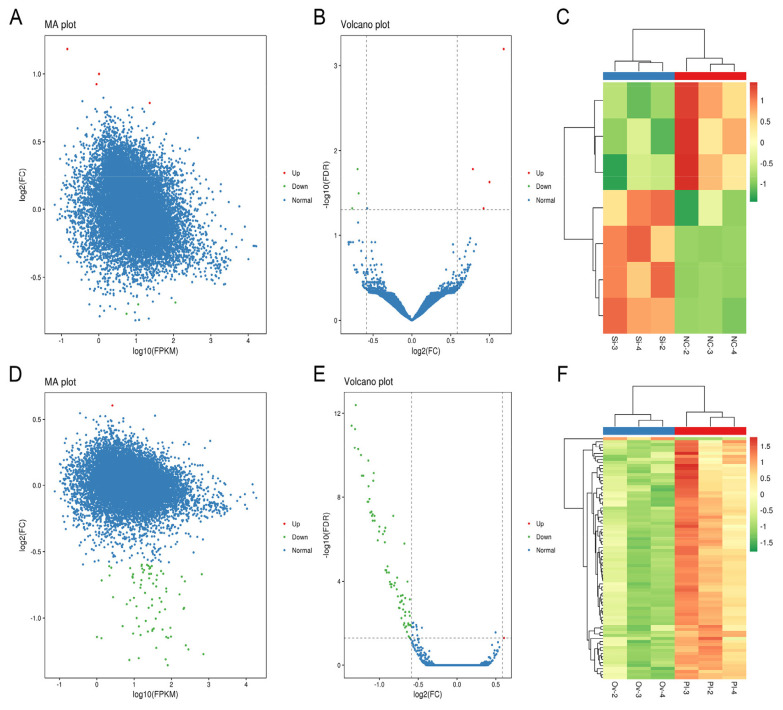
Differential expression analysis of genes in circCOL1A1-knockdown and circCOL1A1-overexpressing goat hair follicle stem cells (gHSFCs). (A, B) MA plot and Volcano plot of 7 differentially expressed genes (DE-genes) in circCOL1A1-knockdown gHFSCs selected based on log2_FC≥0.5 and FDR-value<0.05. (C) Hierarchical clustering plot of 7 DE-genes in the NC group vs. the SI group. (D, E) MA plot and Volcano plot of 80 differentially expressed genes (DE-genes) in circCOL1A1-overexpressing gHFSCs selected based on log2_FC≥0.5 and FDR-value<0.05. (F) Hierarchical clustering plot of 80 DE-genes in the PL group vs. the OV group. NC: the negative control of SI; SI, the circCOL1A1-si; PL, the negative control of Over; OV, the circCOL1A1 overexpression; log2_FC, the fold change of gene expression; FDR, false discovery rate.

In all, 7 DE-genes were found in circCOL1A1-knockdown gHFSCs (NC-groups-vs- SI-groups). The 4 up-regulated genes are shown in red in the MA and volcano plots, the 3 down-regulated genes are shown in green (Figures 1A, 1B), and a hierarchical clustering heatmap of the 7 DE-genes in circCOL1A1-knockdown gHFSCs is also shown (Figure 1C). 80 DE genes were found in circCOL1A1-overexpressing gHFSCs (PL-groups-vs-OV-groups). The 1 up-regulated gene (*CMTM3* gene) is shown in red in the MA plot and volcano plots, the 79 down-regulated genes are shown in green (Figures 1D, 1E), and the 80 DE-genes hierarchical clustering heatmaps of circCOL1A1-overexpressing gHFSCs are also shown (Figure 1F). Among these DE-genes, 7 DE-genes in the NC-vs-SI groups and 9 DE-genes (1 up-regulated and top 10 most down-regulated) in the PL-vs-OV groups were selected and are listed in Tables 1, 2.

**Figure 2:**
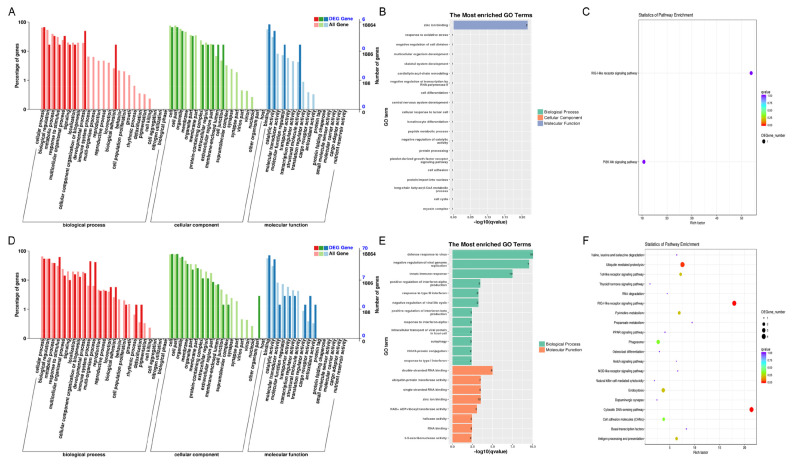
Functional enrichment analysis of DE-genes in circCOL1A1-knockdown and circCOL1A1-overexpressing goat hair follicle stem cells (gHSFCs). (A) Go terms enriched among DE-genes in circCOL1A1-knockdown gHFSCs (NC vs. SI). (B) The most enriched Go terms of DE-genes in the NC group vs. the SI group. (C) The KEGG analysis of DE-genes in the NC group vs. the SI group. (D) Go terms enriched among DE-genes in circCOL1A1-overexpressing gHFSCs (PL vs. OV). (E) The most enrich Go terms of DE-genes in the PL group vs. the OV group. (F) The KEGG analysis of DE-genes in the PL group vs. the OV group. GO, Gene Ontology; NC, the negative control of SI; SI, the circCOL1A1-si; PL, the negative control of over; OV, the circCOL1A1 overexpression; KEGG, Kyoto encyclopedia of genes and genomes.

GO and KEGG analyses were used to examine the DE-genes functions. In NC-vs-SI groups, among the BP terms, development process, adhesion, and cellular activities were enriched. Among CC terms, cell and cell portion, organelle and organelle part, and cell junction part were mainly enriched. Among the MF terms, interaction, catalytic and structural molecule activity were the most enriched (Figure 2A). Additionally, zinc ion binding was the most enriched GO term in MF category (Figure 2B). Furthermore, KEGG assessment showed that the DE-genes revealed largest enrichment in the RIG-I-like receptor and PI3K-Akt signaling pathways (Figure 2C). In PL-vs-OV groups, among the BP terms, response to stimulus, immune system and multi-organism processes were enriched. Among CC terms, in addition to the cell and cell portion, organelle and organelle part, the membrane part was also mostly enriched. Among the MF terms, besides to the interaction, as well as catalytic and antioxidant activities were also mostly enriched (Figure 2D). In addition, defense response to virus, innate immune response, negative viral genome replication modulation, zinc ion binding, and ubiquitin-protein transferase activity showed the most enrichment in the BP and MF categories (Figure 2E). KEGG analysis revealed that these DE-genes were enriched mostly in the Ubiquitin mediated proteolysis, RIG-I-like receptor and cytosolic DNA-sensing signaling pathways (Figure 2F). Based on these findings, the DE-genes are primarily associated with the cellular immune system response, ubiquitin mediation, and cell growth and development.

### DE-miRNAs analysis in all treated gHFSCs samples

**Figure 3:**
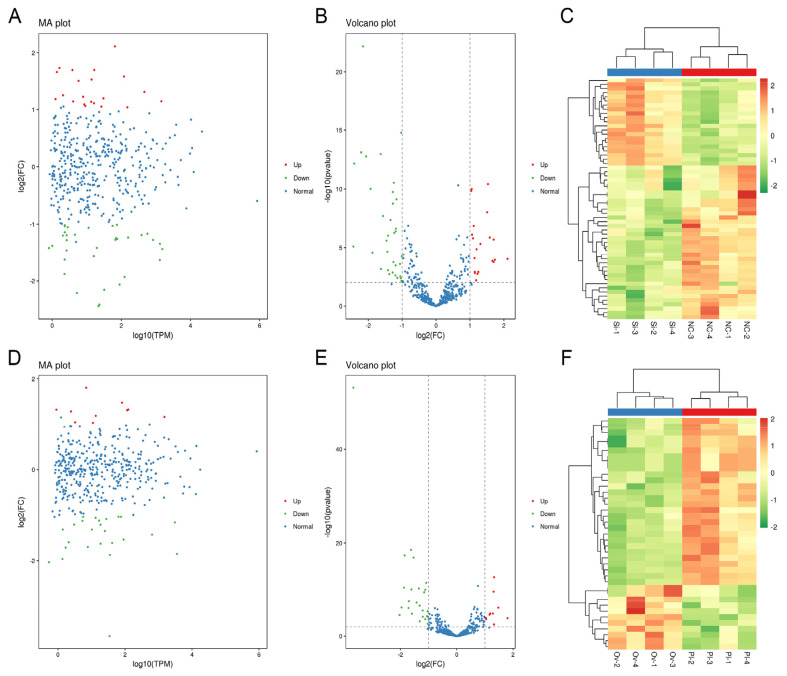
Differential expression analysis of microRNAs (miRNAs) in circCOL1A1-knockdown and circCOL1A1-overexpressing goat hair follicle stem cells (gHSFCs). (A, B) MA plot and Volcano plot of 58 differentially expressed miRNAs (DE-miRNAs) in circCOL1A1-knockdown gHFSCs, selected according to log2_FC ≥ 1.0 and FDR-value < 0.01. (C) Hierarchical clustering plot of 58 DE-miRNAs in the NC group vs. the SI group. (D, E) MA plot and Volcano plot of 38 differentially expressed miRNAs (DE-miRNAs) in circCOL1A1-overexpressing gHFSCs, selected according to log2_FC ≥ 1.0 and FDR-value < 0.01. (F) Hierarchical clustering plot of 38 DE-miRNAs in the PL group vs. the OV group. NC, the negative control of SI; SI, the circCOL1A1-si; PL, the negative control of Over; OV, the circCOL1A1 overexpression; log2_FC, the fold change of gene expression; FDR, false discovery rate.

In all, 58 DE-miRNAs, were found in circCOL1A1-knockdown gHFSCs (NC-groups-vs -SI-groups). The 21 up-regulated miRNAs (red) and 37 down-regulated miRNAs (green) are provided in the MA and volcano plots (Figures 3A, 3B), and a hierarchical clustering heatmap of the 58 DE-miRNAs in circCOL1A1-knockdown gHFSCs is also shown (Figure 3C). 38 DE miRNAs were found in circCOL1A1-overexpressing gHFSCs (PL-groups-versus-OV-groups). The 11 up-regulated miRNAs (red) and 27 down-regulated (including chi-miR-149-5p) miRNAs (green) are provided in the MA and volcano plots (Figures 3D, 3E), and 38 DE-miRNAs hierarchical clustering heatmaps in circCOL1A1- overexpressing gHFSCs are also shown (Figure 3F). Among these DE-miRNAs, 20 DE-miRNAs (top 10 most up- and down-regulated) in NC-vs-SI groups and 20 DE-miRNAs (top 10 most up- and down-regulated) in PL-vs-OV groups were selected and are listed in Tables 3, 4.

**Figure 4:**
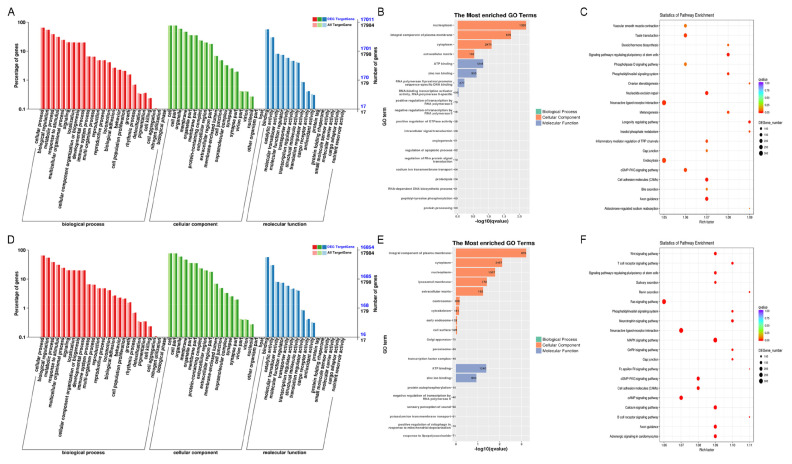
Functional enrichment analysis of DE-miRNAs in circCOL1A1-knockdown and circCOL1A1-overexpressing goat hair follicle stem cells (gHSFCs). (A) Go terms of DE-miRNAs target-genes in circCOL1A1-knockdown gHFSCs (NC vs. SI). (B) The most enriched Go terms of DE-miRNAs in the NC group vs. the SI group. (C) The KEGG analysis of DE-miRNAs target-genes in the NC group vs. the SI group. (D) Go terms of DE-miRNAs target-genes in circCOL1A1-overexpressing gHFSCs (PL vs. OV). (E) The most enriched Go terms of DE-miRNAs in the PL group vs. the OV group. (F) The KEGG analysis of DE-miRNAs target-genes in the PL group vs. the OV group. GO, Gene Ontology; DE, differentially expressed; NC, the negative control of SI; SI, the circCOL1A1-si; KEGG, Kyoto encyclopedia of genes and genomes; PL, the negative control of Over; OV, the circCOL1A1 overexpression.

Most miRNAs function by regulating target genes, resulting in changes of or silencing of target genes expression. GO and KEGG assessments were used to focus on investigating the DE-miRNAs target gene functions. In NC-vs-SI groups and PL-vs-OV groups, for the BP, cellular process, biological modulation, and metabolic process were mainly enriched. For the CC, cell and cell portion, organelle and membrane were mainly enriched. For the MF, binding and catalytic activity were mainly enriched (Figures 4A, 4D). In addition, in both the NC-vs-SI and PL-vs-OV groups, nucleoplasm, integral component of plasma membrane, cytoplasm, extracellular matrix and adenosine triphosphate (ATP) interaction, zinc ion association in CC and MF GO terms were the most enriched (Figures 4B, 4E). Furthermore, KEGG analysis showed that DE-miRNAs target genes in NC-vs-SI groups were enriched mostly in neuroactive ligand-receptor interaction, endocytosis, and cell adhesion molecules (Figure 4C). In PL-vs-OV groups, KEGG analysis showed that DE-miRNAs target genes were enriched mostly in Ras and MAPK signaling networks, neuroactive ligand-receptor association, and cAMP signaling pathway (Figure 4F). These evidences indicate that DE-miRNAs and target genes are associated primarily with receptor interactions, molecular absorption, membrane structure composition, cellular development, and transcription factor regulation.

### Differentially expressed-circRNAs analysis in all treated gHFSCs samples

**Figure 5:**
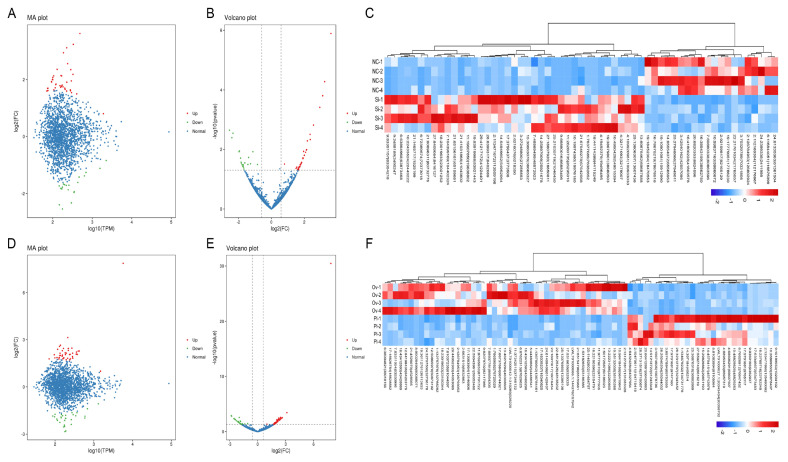
Differential expression analysis of circular RNAs (circRNAs) in circCOL1A1-knockdown and circCOL1A1-overexpressing goat hair follicle stem cells (gHSFCs). (A, B) MA plot and Volcano plot of 59 differentially expressed circRNAs (DE-circRNAs) in circCOL1A1-knockdown gHFSCs selected based on log2_FC ≥ 1.5 and FDR-value < 0.05. (C) Hierarchical clustering plot of 59 DE-circRNAs in the NC group vs. the SI group. (D, E) MA plot and Volcano plot of 76 differentially expressed circRNAs (DE-circRNAs) in circCOL1A1-overexpressing gHFSCs selected based on log2_FC ≥ 1.5 and FDR-value < 0.01. (F) Hierarchical clustering plot of 76 DE-circRNAs in the PL group vs. the OV group. NC, the negative control of SI; SI, the circCOL1A1-si; OV, the circCOL1A1 overexpression; PL, the negative control of Over; log2_FC, the fold change of gene expression; FDR, false discovery rate.

In all, 59 DE-circRNAs were found in circCOL1A1-knockdown gHFSCs (NC-groups-vs -SI-groups). The 39 up-regulated circRNAs (red) and 20 down-regulated cirrcRNAs (green) are provided in the MA and volcano plots (Figures 5A, 5B), and a hierarchical clustering heatmap of the 59 DE-circRNAs in circCOL1A1-knockdown gHFSCs is also displayed (Figure 5C). 76 DE circRNAs were found in circCOL1A1-overexpressing gHFSCs (PL-groups-vs-OV-groups). The 47 up-regulated (including circCOL1A1) circRNAs (red) and 29 down-regulated circRNAs (green) are provided in the MA and volcano plots (Figures 5D, 5E), and 76 DE-circRNAs hierarchical clustering heatmaps in circCOL1A1-overexpressing gHFSCs are also displayed (Figure 5F). Among these DE-circRNAs, 20 DE-circRNAs (top 10 most up- and down-regulated) in NC-vs-SI groups and 20 DE-circRNAs (top 10 most up- and down-regulated) in PL-vs-OV groups were selected and are listed in Tables 5, 6.

**Figure 6:**
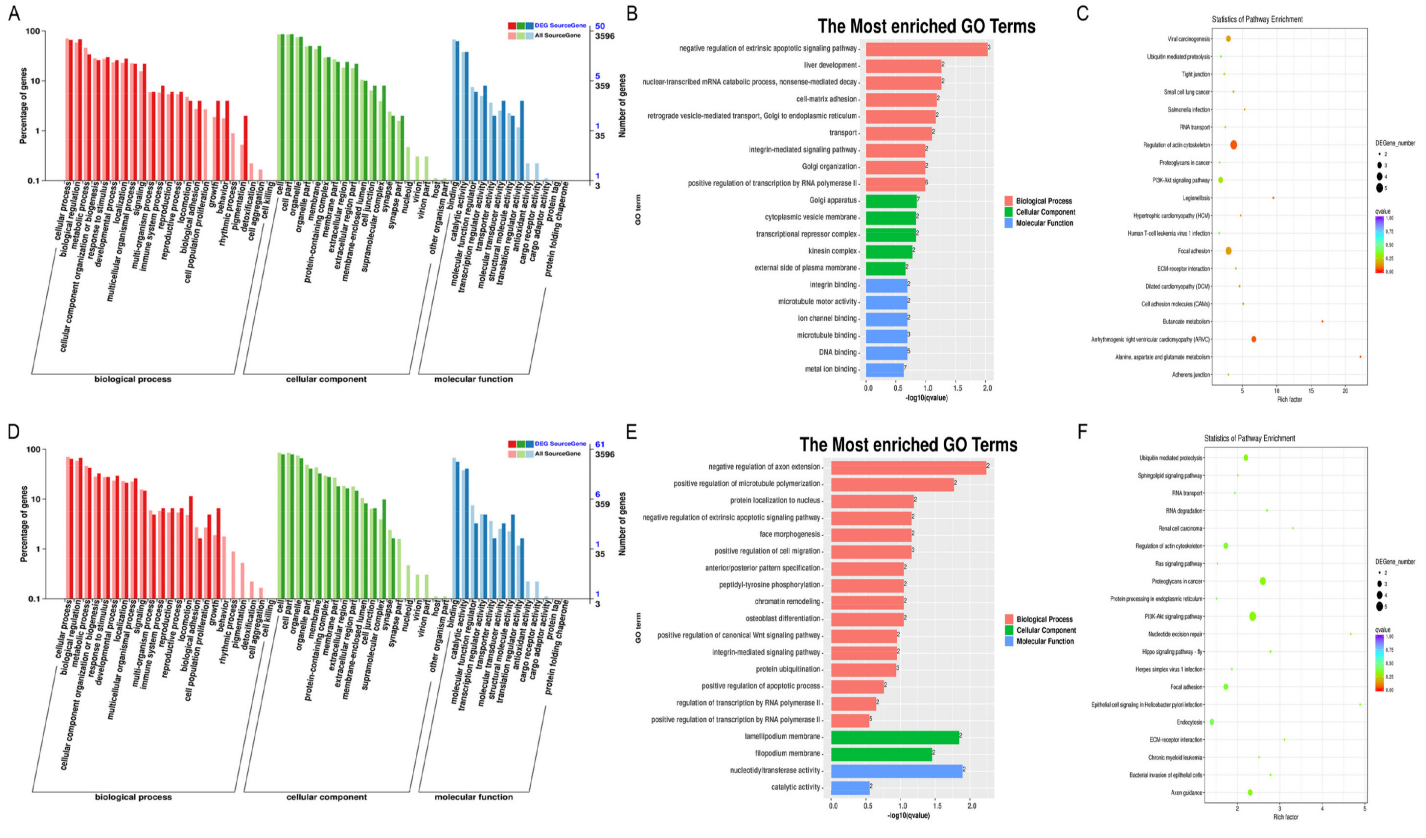
Functional enrichment analysis of DE-circRNAs in circCOL1A1-knockdown and circCOL1A1-overexpressing goat hair follicle stem cells (gHSFCs). (A) Go terms of DE-circRNAs source-genes in circCOL1A1-knockdown gHFSCs (NC vs. SI). (B) The most enriched Go terms of DE-circRNAs in the NC group vs. the SI group. (C) The KEGG analysis of DE-circRNAs source-genes in the NC group vs. the SI group. (D) Go terms of DE-circRNAs source-genes in circCOL1A1-overexpressing gHFSCs (PL vs. OV). (E) The most enriched Go terms of DE-circRNAs in the PL group vs. the OV group. (F) The KEGG analysis of DE-circRNAs source-genes in the PL group vs. the OV group. GO, Gene Ontology; DE, differentially expressed; NC, the negative control of SI; SI, the circCOL1A1-si; KEGG, Kyoto encyclopedia of genes and genomes; PL, the negative control of Over; OV, the circCOL1A1 overexpression.

Most circRNAs in all treated gHFSCs samples were derived from exons of the source genes (protein-coding genes), and it is thought that circRNAs processing can influence precursor transcript splicing in source genes, resulting in changes of source genes level. Therefore, GO and KEGG assessment were used to examine the DE-circRNAs source gene functions. In NC-vs-SI groups and PL-vs-OV groups, for the BP, cellular process, biological modulation, and metabolic process were mainly enriched. For the CC, cell and cell portion, organelle and membrane were mainly enriched. For the MF, binding and catalytic function were mainly enriched (Figures 6A, 6D). Overall, our findings from the GO terms assessment of DE-circRNAs source genes were consistent with those of the DE-miRNAs target genes. In addition, in both the NC-vs-SI and PL-vs-OV groups, direct RNA polymerase II-mediated transcription regulation, integrin-linked pathways, and post modulation of canonical Wnt signaling pathway were the most enriched GO terms (Figures 6B, 6E). Furthermore, in NC-vs-SI groups, KEGG network assessment revealed that DE-circRNAs source genes showed marked enrichment in the actin cytoskeleton regulation, focal adhesion, and PI3K-Akt pathways (Figure 6C). In PL-vs-OV groups, KEGG network assessment revealed that DE-circRNAs source genes were markedly enriched in the PI3K-Akt and focal adhesion signaling pathway (Figure 6F). Based on these findings, the DE-circRNAs source genes likely contribute to the extracellular matrix structures and interactions, gHFSCs growth and development, HF morphogenesis, and the cytoskeleton.

### Interactive network analysis of differentially expressed-genes, differentially expressed-miRNAs, and differentially expressed-circRNAs

**Figure 7:**
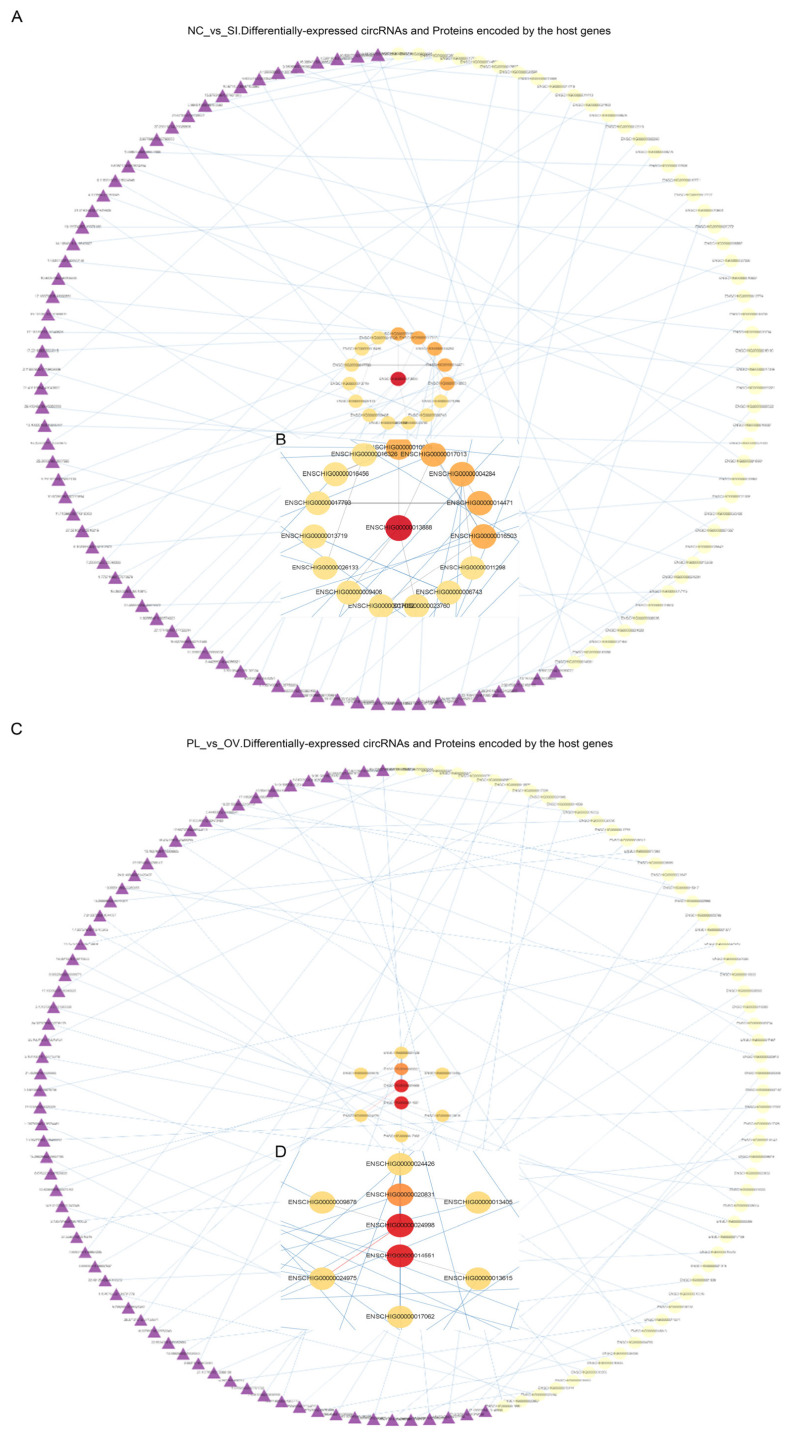
Protein interaction network analysis of DE-circRNAs. (A, B) Interaction network maps of DE-circRNAs, source-genes and proteins in circCOL1A1-knockdown gHFSCs. (C, D) Interaction network maps of DE-circRNAs, source-genes and proteins in circCOL1A1- overexpressing gHFSCs. NC, the negative control of SI; SI, the circCOL1A1-si; PL, the negative control of Over; OV, the circCOL1A1 overexpression; DE, differentially expressed; gHSFCs, goat hair follicle stem cells.

To accurately identify the roles of DE-genes, DE-miRNAs, and DE-circRNAs, two interaction networks among the DE-circRNAs, source genes and proteins were first generated via the STRING protein interaction tool (https://cn.string-db.org/). In NC-vs-SI groups, DE-circRNAs interacted mainly with the proteins coded from source genes, including ENSCH I00000013888, −17013, −04284, −14471, −16503 etc., especially with ENSCHI00000013888 (GEN1, an endonuclease) (Figures 7A, 7B). In PL-vs-OV groups, DE-circRNAs interacted mainly with the proteins encoded by the source genes, including ENSCHI00000024998, −14551, −20831, −09878, −24426 etc., and especially with ENSCHI00000024998 (MET, receptor tyrosine kinase) and ENSCHI00000014551 (ERCC3, an ATP-dependent DNA helicase) (Figures 7C, 7D).

**Figure 8:**
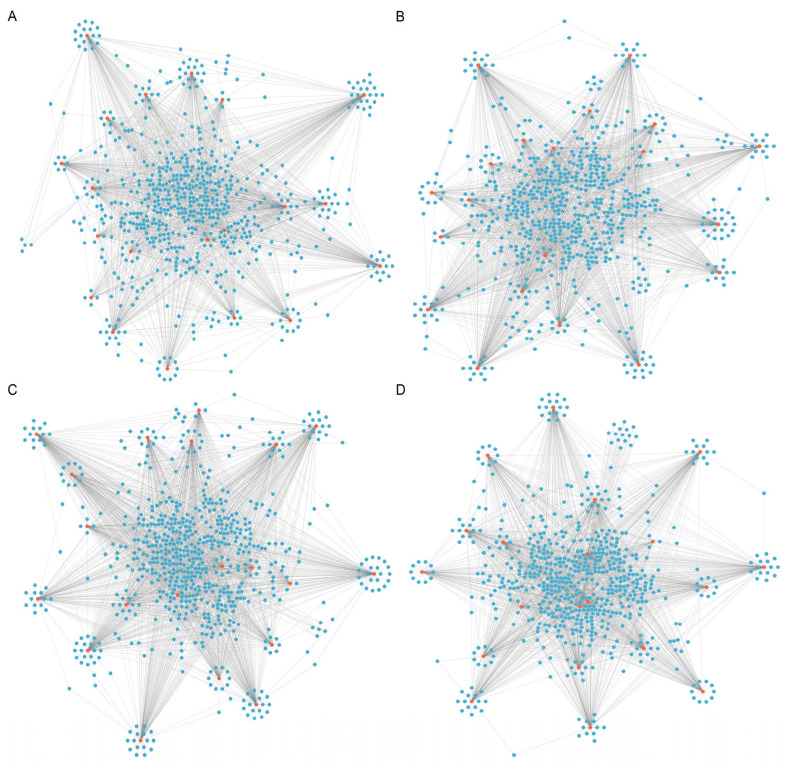
Interactive network analysis of DE-circRNAs and miRNAs. (A) Diagram of the top 20 DE-circRNAs and target miRNAs interaction networks up-regulated in the NC group vs. the SI group. (B) Diagram of the top 20 DE-circRNAs and target miRNAs interaction networks down-regulated in the NC group vs. the SI group. (C) Diagram of the top 20 DE-circRNAs and target miRNAs interaction networks up-regulated in the PL group vs. the OV group. (D) Diagram of the top 20 DE-circRNAs and target miRNAs interaction networks down-regulated in the PL group vs. the OV group. DE, differentially expressed; NC, the negative control of SI; SI, the circCOL1A1-si; PL, the negative control of Over; OV, the circCOL1A1 overexpression.

In addition, via conjoint analysis of the miRNA-seq data and circRNA-seq data with RNAhybrid (*v*2.1.2), miRanda (*v*3.3a), TargetScan (https://www.targetscan.org/vert_72/) databases, we constructed DE-circRNAs-miRNAs sponged interaction networks. The top 20 up-regulated DE-circRNAs and the top 20 down-regulated DE-circRNAs in NC-vs-SI groups, with their target miRNAs sponging networks are shown in Figures 8A, 8B. The top 20 up-regulated DE-circRNAs and the top 20 down-regulated DE-circRNAs in PL-vs-OV groups, with their target miRNAs sponging networks are shown in shown in Figures 8C, 8D. In these sponge interaction axes, the red nodes indicate the up- or down-regulated DE-circRNAs, and the blue nodes indicate each miRNA targeted by DE-circRNAs.

**Figure 9:**
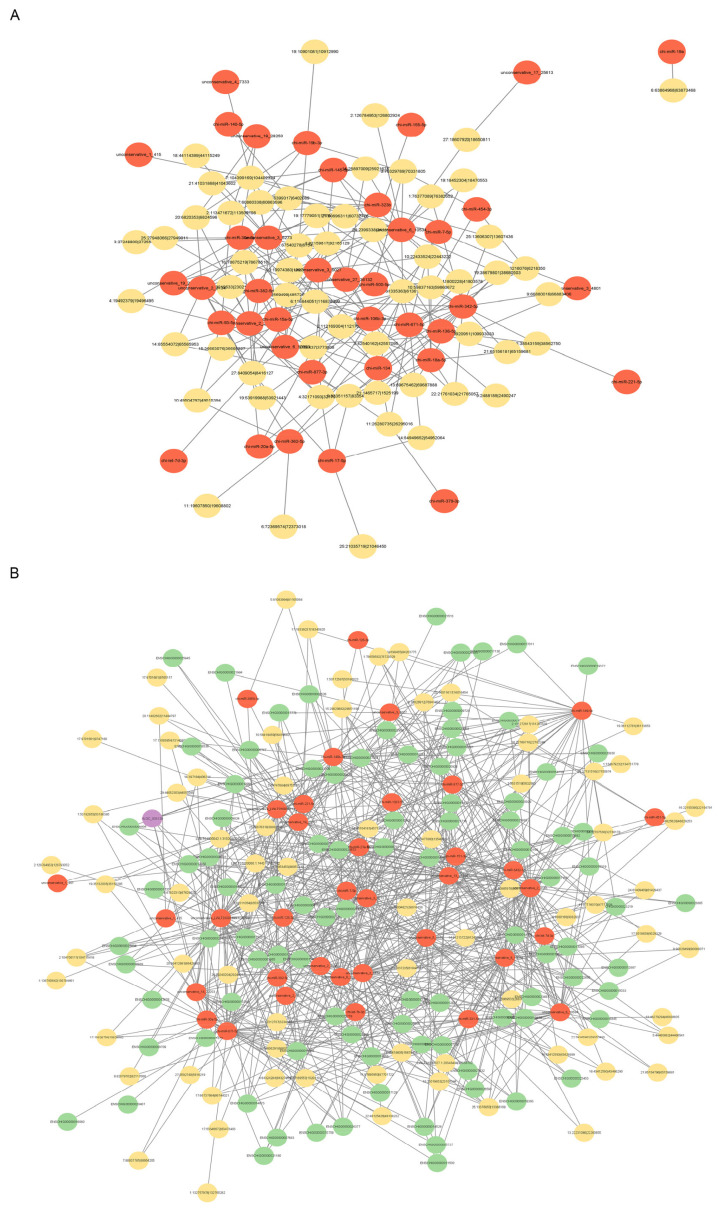
Interactive network relationships between DE-circRNAs, miRNAs and genes. (A) Competing endogenous RNAs (ceRNAs) network of DE-circRNAs-miRNAs-genes in circCOL1A1-knockdown gHFSCs (NC vs. SI). (B) Competing endogenous RNAs (ceRNAs) network of DE-circRNAs-miRNAs-genes in circCOL1A1-overexpressing gHFSCs (PL vs. OV). DE, differentially expressed; gHSFCs, goat hair follicle stem cells; NC, the negative control of SI; SI, the circCOL1A1-si; PL, the negative control of Over; OV, the circCOL1A1 overexpression.

Moreover, conjoint analysis and systematic clustering methods were used to analyze the gene-seq data, miRNA-seq data and circRNA-seq data to construct the ceRNAs networks between them. As shown in Figure 9A, in the NC-vs-SI groups, the ceRNA axes of DE-genes, DE-miRNAs, and DE-circRNAs, such as chi-miR-145-5p, a down-regulated miRNA, was targeted by multiple DE-circRNAs, including circUPF1 (7:104399169|104402324), circSTAM2 (2:92159817|92165129), circACTN1 (10:22433524|22443222) and circSTRN3 (21:41031868|41043602). In PL-vs-OV groups, the ceRNA axes of DE-genes, DE-miRNAs, and DE-circRNAs, such as chi-miR-451-5p, the most up-regulated miRNA, which was targeted by multiple DE-circRNAs, including circMTARC2 (16:22769716|22782249), circCDYL (23:16011617 |16016454), and circLCORL (6:38004821|38018156) are shown in Figure 9B. Moreover, the chi-miR-451-5p could also target multiple genes, including ENSCHI00000015319 (*PKDREJ*), −19892 (*OAS1*) and −10163 (a novel gene). Besides, the known ceRNA network circCOL1A1-miR-149-5p-CMTM3 was found among these networks (19:36112781| 36113653-chi-miR-149-5p-ENSCHI00000019372). Additionally, chi-miR-149-5p could also be targeted by other DE-circRNAs, including circDIAPH3 (12:84198435|84203775) and circKNTC1 (17:18339237| 18340825); it could also target the other genes in addition to *CMTM3*, such as ENSCHIG00000017136 (*RNF213*), −14238 (*IFIT3*) and −16319 (*EIF2AK2*).

### Identification and validation of differentially expressed-genes, differentially expressed-miRNAs, and differentially expressed-circRNAs

**Figure 10:**
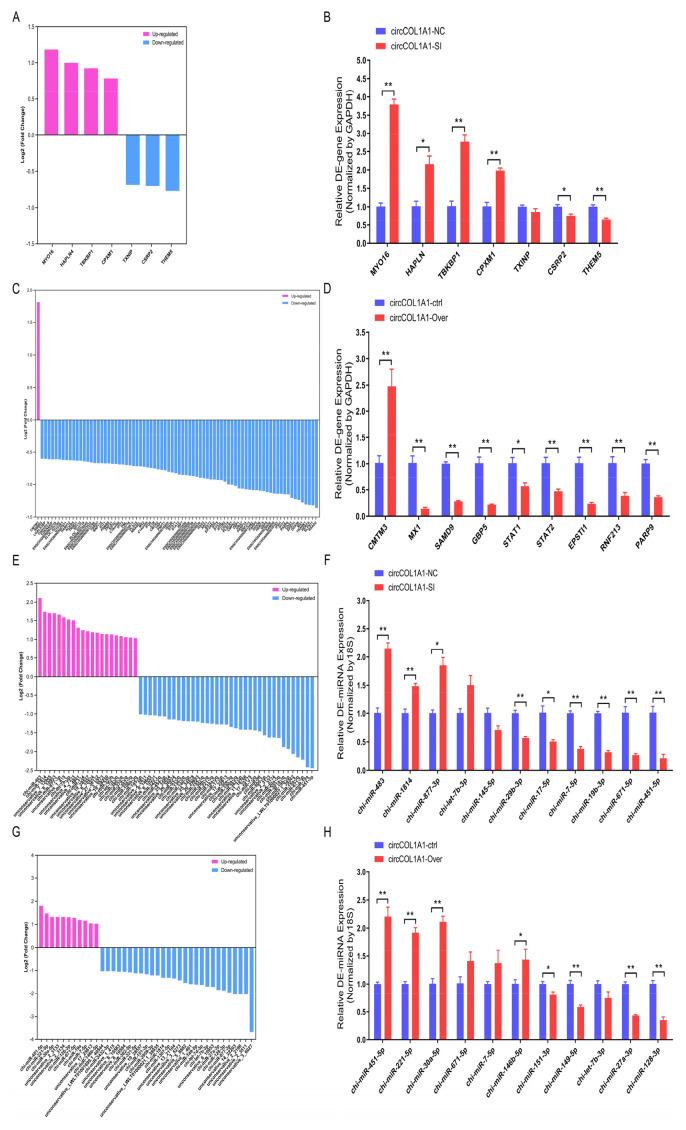
Identification and validation of differentially expressed genes and miRNAs. (A) The log2_fold change values of 7 DE-genes in circCOL1A1-knockdown gHFSCs (NC vs. SI). (B) The expression levels of 7 DE-genes in circCOL1A1-knockdown gHFSCs were further verified in triplicate by RT-qPCR assay. (C) The log2_fold change values of 80 DE-genes in circCOL1A1-overexpressing gHFSCs (PL vs. OV). (D) The expression levels of 9 randomly selected DE-genes expression levels in circCOL1A1-overexpressing gHFSCs were further verified in triplicate by RT-qPCR assay. (E) The log2_fold change values of 58 DE-miRNAs in circCOL1A1-knockdown gHFSCs (NC vs. SI). (F) The expression levels of 11 randomly selected DE-miRNAs in circCOL1A1-knockdown gHFSCs were further verified in triplicate by RT-qPCR assay (G) The log2_fold change values of 38 DE-genes in circCOL1A1-overexpressing gHFSCs (PL vs. OV). (H) The expression levels of 11 randomly selected DE-miRNAs in circCOL1A1-overexpressing gHFSCs were further verified in triplicate by RT-qPCR assay. * p<0.05 and ** p<0.01. NC, the negative control of SI; SI, the circCOL1A1-si; log2_FC, the fold change of gene expression; DE, differentially expressed; gHSFCs, goat hair follicle stem cells; RT-qPCR, real-time quantitative polymerase chain reaction; PL, the negative control of Over; OV, the circCOL1A1 overexpression.

**Figure 11:**
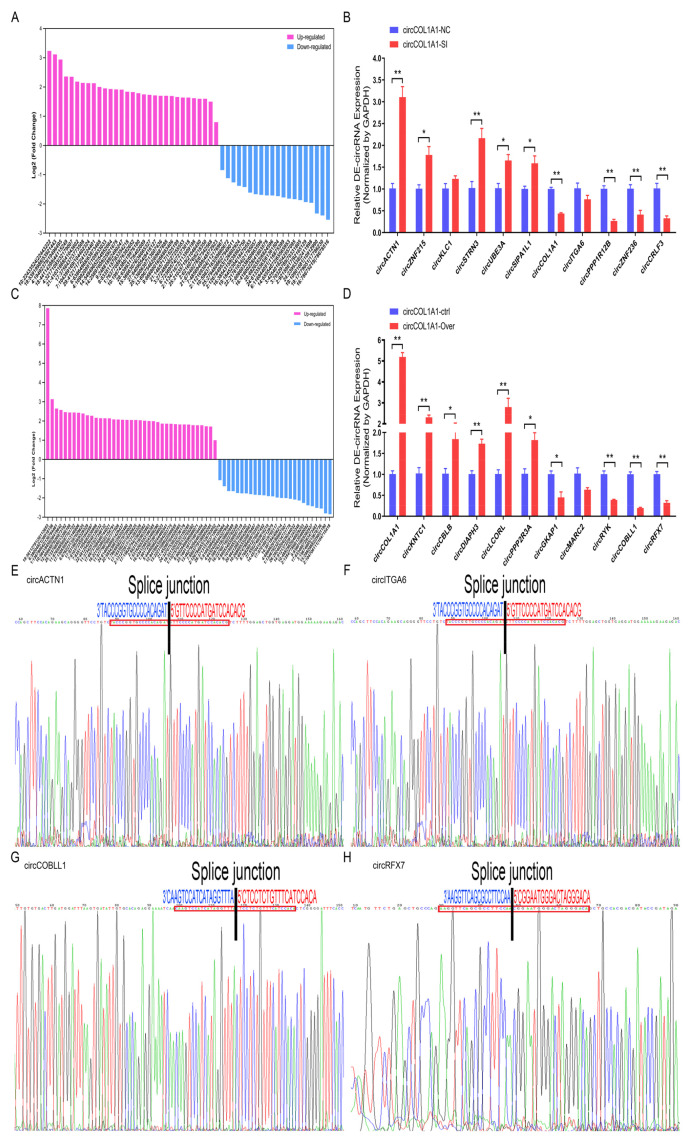
Identification and validation of differentially expressed circRNAs. (A) The log2_fold change values of 59 DE-circRNAs in circCOL1A1-knockdown gHFSCs (NC vs. SI). (B) 11 randomly selected DE-circRNAs expression levels in circCOL1A1-knockdown gHFSCs were further verified in triplicate by RT-qPCR assay. (C) The log2_fold change values of 76 DE-circRNAs in circCOL1A1-overexpressing gHFSCs (PL vs. OV). (D) 11 randomly selected DE-circRNAs expression levels in circCOL1A1-overexpressing gHFSCs were further verified in triplicate by RT-qPCR assay. (E) The splice junction site sequence of circACTN1 was confirmed via Sanger sequence. (F) The splice junction site sequence of circITGA6 was confirmed via Sanger sequence. (G) The splice junction site sequence of circCOBLL1 was confirmed via Sanger sequence. (H) The splice junction site sequence of circRFX7 was confirmed via Sanger sequence. * p<0.05 and ** p<0.01. DE, differentially expressed; NC, the negative control of SI; SI, the circCOL1A1-si; log2_FC, the fold change of gene expression; gHSFCs, goat hair follicle stem cells; RT-qPCR, real-time quantitative polymerase chain reaction; PL, the negative control of Over; OV, the circCOL1A1 overexpression.

To further verify the reliability and authenticity of the whole-transcriptome sequencing results, a total of 7 up-regulated or down-regulated genes, 11 up-regulated or down-regulated miRNAs, 11 up-regulated or down-regulated circRNAs from NC-vs-SI groups were chosen for RT-qPCR assay; A total of 9 up-regulated or down-regulated genes, 11 up-regulated or down-regulated miRNAs, 11 up-regulated or down-regulated circRNAs from PL-vs-OV groups were selected for RT-qPCR assay. As measured by RT-qPCR, the 7-DE genes expression tendencies (such as *MYO16* gene) in NC-vs-SI groups were consistent with our gene-seq results of whole-transcriptome sequencing (Figures 10A, 10B); the expression tendencies of 9 DE-genes (such as *CMTM3* gene) in PL-vs-OV groups were also consistent with our gene-seq results of whole-transcriptome sequencing (Figures 10C, 10D). For DE-miRNAs, the expression tendencies of 11 DE-miRNAs (such as chi-let-7b-3p and chi-miR-671-5p) in NC-vs-SI groups (Figures 10E, 10F), and 11 DE-miRNAs (such as chi-miR-451-5p and chi-miR-149-5p) in PL-vs-OV groups (Figures 10G, 10H) were consistent with our miRNAs whole-transcriptome sequencing results. For DE-circRNAs, the expression tendencies of 11 DE-circRNAs (such as circACTN1 and circCOL1A1) in NC-vs-SI groups were consistent with our whole-transcriptome circRNA-seq results (Figures 11A, 11B); the expression tendencies of 11 DE-circRNAs (such as circCOL1A1 and circGKAP1) in PL-vs-OV groups were consistent with our whole-transcriptome circRNA-seq results too (Figures 11C, 11D). In addition, four DE-circRNAs (circACTN1, circITGA6, circCOBLL1, and circRFX7) were further selected for splicing site (also called junction site) identification via Sanger sequencing; the splicing sites of circACTN1 (Figure 11E), circITGA6 (Figure 11F), circCOBLL1 (Figure 11G), circRFX7 (Figure 11H) were verified, and the sequences information was consistent with our whole-transcriptome circRNA-seq results. These results suggest that our whole-transcriptome sequencing results are credible.

## DISCUSSION

SQBH is the most important economic product of YRDWG, and SQBH formation is tightly related to the YRDWG gHFSCs growth and HF development. Deeply clarifying the regulatory mechanism of YRDWG gHFSCs growth and HF development is critical for explaining the SQBH formation process and improving its yield. Recently, an increasing number of studies have revealed the regulatory mechanism by which miRNAs /lncRNAs/circRNAs and genes affect cashmere goats HF development and cashmere generation. For example, via the use of lncRNA-sequencing technology in Chinese cashmere goats (Liaoning cashmere goats vs. Ziwuling cashmere goats), totally 129 DE lncRNAs were obtained in these two cashmeres goats’ caprine skins [30]. These lncRNAs’ target genes are associated with the color, diameter, and synthesis of cashmere, which reveals the role of lncRNAs in Chinese cashmere goat fiber formation. By combining RNA-sequencing and miRNA-sequencing in Inner Mongolian Arbas white cashmere goats, the *FZD6*, *SMAD2*, *WNT5a*, and *LEF1* genes were found to be DE in growing and resting stage skin tissues. In addition, these genes are regulated by miR-195, miR-148a, and miR-335 and participate in the Wnt/β-catenin signaling pathway [31]. By analyzing whole-transcriptome sequencing data from Arbas white cashmere goats skin tissues, 6468 DE-genes, 394 DE-miRNAs, and 239 DE-circRNAs were discovered, and multiple ceRNA-regulatory networks were constructed, including the chi-circRNA0001141-miR-184-FGF10 network [32]. Using both sequencing and weighted gene co-expression network analysis, which included *COL1A1*, *COL1A2*, *KRTAP3-1*, *FA2H*, etc., 12 genes were obtained and were significantly related to HF development; in addition, the ECM-receptor interaction, focal adhesion, PI3K/Akt and MAPK signaling pathways were the main pathway related to HF growth and development [33]. These studies suggest that DE genes, miRNAs, lncRNAs or circRNAs play different roles and functions in the development of HF in cashmere goats.

However, none of these are associated with the YRDWG gHFSCs growth or HF development. Our previous studies indicated that the circCOL1A1-miR-149-5p-CMTM3/AR network plays important roles in modulating gHFSCs growth, HF development, and SQBH formation [6,7,12]. Herein, based on this evidence, we constructed circCOL1A1-overexpressing and circCOL1A1-knockdown YRDWG gHFSCs and investigated the regulatory patterns and interaction networks between genes and noncoding RNAs through via whole-transcriptome sequencing. After genes, miRNAs, and circRNAs sequencing data were analyzed, we obtained 7, 58, and 59 DE-genes, DE-miRNAs, and DE-circRNAs, respectively, in circCOL1A1-knockdown YRDWG gHFSCs; and 80, 38, and 76 DE-genes, DE-miRNAs, and DE-circRNAs, respectively, in circCOL1A1-overexpressing YRDWG gHFSCs. GO annotation and KEGG assessments uncovered that these DE-genes, DE-miRNAs, and DE-circRNAs showed most enrichment in the PI3K/Akt and MAPK signaling pathways, focal adhesion, regulating pluripotency of stem cells, the Wnt signaling pathway, ECM-receptor interaction, and cellular physiological processes etc. Then, approximately 11 DE genes, miRNAs, and circRNAs were randomly selected in circCOL1A1-overexpressing or circCOL1A1-knockdown YRDWG gHFSCs to confirm the expression differences via RT-qPCR and Sanger sequencing. We demonstrated that these selected genes, miRNAs, and circRNAs expression profiles in whole-transcriptome sequencing and RT-qPCR closely mirrored one another, further indicating both reliability and precision of the YRDWG gHFSCs sequencing.

For DE genes in NC-vs-SI groups, MYO16 is the most up-regulated gene after circCOL1A1 was knocked down in the YRDWG gHFSCs. MYO16 encodes an unconventional myosin that serves as a serine/threonine phosphatase-1 targeting and modulatory subunit. Tyrosine-site phosphorylated MYO16 associates with the p85 regulatory subunit of PI3K and then participates in PI3K/Akt signaling; besides, the interaction between MYO16 and NYAP3 has been verified to contribute to the PI3K/Akt signaling pathway mediated the remodeling of the actin cytoskeleton [34,35]. In agreement with results of these results, KEGG and GO analyses of our seq-data showed that the MYO16 gene is also enriched mostly in the PI3K/Akt signaling pathway, which means that MYO16 is tightly associated with YRDWG gHFSCs growth and HF development. For DE genes in PL-vs-OV groups, CMTM3 is the most up-regulated gene after circCOL1A1 overexpression in YRDWG gHFSCs, which is in accordance with our previous studies, and the CMTM3 expression level is regulated by circCOL1A1 expression in YRDWG skin tissue and gHFSCs [12]. In addition, the STAT1 and STAT2 are down-regulated in in PL-vs-OV groups. The STAT1 and STAT2 genes encode the corresponding proteins that are key members of the STAT protein family. STAT1 and STAT2 can be phosphorylated by receptor-associated kinases such as JAK (Janus kinase) and MAPK (mitogen-activated protein kinase), which induces homo- or heterodimer formation, which then transfers to the cell nucleus to serve as transcription activators (namely, the JAK-STAT and MAPK-ERK pathways) [36]. KEGG and GO analyses of our sequencing-data showed that STAT1 and STAT2 are annotated in the innate immune response and response to type-I/III interferon processes. MAPK controls STAT1 and STAT2 expression and activation to participate in MAPK signaling pathway, which further suggested that the roles of STAT1 and STAT2 depend on the MAPK and MAPK signaling pathway during the gHFSCs growth and HF development of the YRDWG.

For DE miRNAs, chi-let-7b-3p is up-regulated in NC-vs-SI groups and down-regulated in PL-vs-OV groups. On the contrary, chi-miR-451-5p and chi-miR-671-5p are both down-regulated in NC-vs-SI groups and are both up-regulated in PL-vs-OV groups. Besides, chi-miR-149-5p expression was inhibited in circCOL1A1-overexpressing YRDWG gHFSCs; meanwhile, chi-miR-145-5p expression was suppressed in circCOL1A1- knockdown YRDWG gHFSCs. These results indicated that abovementioned DE-miRNAs are potentially sequestered and modulated by circCOL1A1 during the growth of YRDWG gHFSCs. Our previous studies also reported that chi-miR-149-5p regulates YRDWG gHFSCs proliferation and differentiation through targeting the CMTM3/AR axis [6,7] and that chi-miR-145-5p promotes YRDWG gHFSCs differentiation via control the NANOG and Sox9 contents [10]. Moreover, other studies have indicated that fibroblast growth factor 5 (FGF5), transforming growth factor beta receptor I (TGFβR1), and ectodysplasin A can be targeted by let-7b-3p [37]; and subsequently play important roles in the VEGF, TGF-β, and NF-κB signaling pathways in cashmere goats and alpaca hair growth and skin development [38]. To date, there has been no research associating the functions of miR-451-5p and miR-671-5p with gHFSCs growth and HF development. In addition, KEGG and GO analyses revealed that these miRNAs and their target genes are showing marked enrichment in the MAPK, Wnt, and cell adhesion signaling pathways, indicating their importance in YRDWG gHFSCs growth and HF development.

For DE circRNAs, circCOL1A1, as a known functional circRNA, was effectively up- or down-regulated in circCOL1A1-overexpressing and circCOL1A1-knockdown gHFSCs. Through integrated analysis and interaction network construction, we further verified our previous results that the chi-circCOL1A1-miR-149-5p-CMTM3-AR axis. Besides, circACTN1, derived from the source gene α-actinin 1 (ACTN1), is the most up-regulated circRNA in YRDWG gHFSCs after circCOL1A1 knockdown. ACTN1 can interact with ITGA5/6 (integrin alpha 5/6) and subsequently participate in the focal adhesion process [39]. Focal adhesion plays important roles in activating HFSCs and HF development, and is beneficial for HF morphogenesis [40]. circITGA6, derived from the source gene ITGA6, is down-regulated in YRDWG gHFSCs after circCOL1A1 knockdown. The ITAG6 gene encodes the ITGA6 protein, which belongs to the integrin protein superfamily. Integrins are cell adhesion receptors that function in signaling pathways from the extracellular matrix to cells [41]. Our constructed interaction network showed that circACTN1 could sponge chi-miR-106b-3p, chi-miR-671-5p, chi-miR-18a-5p, chi-miR-323b, etc. and that circITGA6 could sponge chi-miR-877-3p, chi-miR-671-5p, chi-miR-342-5p, chi-miR-134, chi-miR-18a-5p, etc. Interestingly, circACTN1 and circITGA6 both sponge the chi-miR-671-5p and chi-miR-18a-5p. Through Functional analysis (using the TargetScan and RNAhybrid databases) and GO annotation, we found that chi-miR-671-5p targets KRTAP3, ITGA2, COL13A1, and MAPK3; while, chi-miR-18a-5p targets FGF1, MAP3K1, and MAPK4. MAPK3/4 [42], FGF1 [43], MAP3K1 [44], ITGA2 [45], KRTAP3 [33], and COL13A1 [46] are reported to function in regulating gHFSCs growth, DPCs differentiation and HF development. Based on these results, we speculated that circACTN1 and circITGA6 are essential and beneficial for gHFSCs growth and HF development in YRDWG via the chi-circACTN1-miR-671-5p-MAPK3/COL13A1 axis, and chi-circITGA6-miR-18a-5p-FGF1 /MAP3K1 axis. circCOBLL1, which originates from the source gene COBLL1, is the most down-regulated circRNA in YRDWG gHFSCs after circCOL1A1 overexpression. COBLL1 (cordon-bleu WH2 repeat protein like 1) gene could enable cadherin binding activity and is localized to the extracellular exosomes. Furthermore, COBLL1 has been identified as related to drug resistance in chronic myeloid leukemia, gestational diabetes, and prostate cancer [47,48]. However, until now, no studies have reported its function in HFSC growth or HF development. Functional analysis and GO annotation showed that circCOBLL1 could sponge chi-miR-30a-5p and chi-miR-128-3p (two down-regulated miRNAs), and ITGA2, ITGA6, MAPK14, FGF20, and FGF14 are all the target genes of these two miRNAs. Therefore, we speculated that the chi-circCOBLL1-miR-30a-5p/miR-128-3p-ITGA6/MAPK14/FGF14 axis might also be critical for YRDWG gHFSCs growth and HF development. Together, combining the above sequencing results, data analysis and reported literature, we selected circACTN1, circITGA6, circCOBLL1 and their interaction networks for our further in-depth research on YRDWG gHFSCs growth, HF development and SQBH formation.

In conclusion, based on our whole-transcriptome sequencing data, we identified 87 DE-genes, 96 DE-miRNAs, and 135 DE-circRNAs in circCOL1A1-knockdown and circCOL1A1- overexpressing YRFWG gHFSCs. Functional enrichment, GO annotation and KEGG assessment uncovered that the CMTM3, MYO16, STAT1, STAT2, chi-let-7b-3p, chi- miR-671-5p, chi-miR-451-5p, chi-miR-149-5p, chi-miR-145-5p, circCOL1A1, circACTN1, circITGA6, and circCOBLL1 could participate in regulating YRDWG gHFSCs growth and HF development. Moreover, mechanism of action and interaction network analysis revealed that chi-circCOL1A1-miR-149-5p-CMTM3-AR, chi-circACTN1-miR-671-5p-MAPK3/ COL13A1, chi-circITGA6-miR-18a-5p-FGF1/MAP3K1 and chi-circCOBLL1-miR-30a-5p/ miR-128-3p-ITGA6/MAPK14/FGF14 axes could play exceedingly important roles in YRDWG gHFSCs growth, HF development and SQBH formation. These novel findings provide a valuable and comprehensive basis for investigating the complex mechanisms regulating skin tissue HF development and SQBH traits formation in YRDWG.

## Figures and Tables

**Table 1:** The differentially expressed genes in circCOL1A1-knockdown goat hair follicle stem cells (gHSFCs)

ID	Gene_name	NC2-FPKM	NC3-FPKM	NC4-FPKM	SI2-FPKM	SI3-FPKM	SI4-FPKM	p-value	FDR	Log2_FC	Regulated
ENSCHIG 00000002331	MYO16	0.028304	0.016947	0.020334	0.218212	0.301259	0.278563	4.63E-08	0.000638	1.182943	Up
ENSCHIG 00000017398	HAPLN4	0.416897	0.395224	0.332793	1.550089	1.515655	1.874257	6.87E-06	0.023679	0.999616	Up
ENSCHIG 00000016860	TBKBP1	0.386434	0.413806	0.416441	1.506712	1.101528	1.381534	2.80E-05	0.048247	0.923772	Up
ENSCHIG 00000021212	CPXM1	19.2219	12.51094	14.54348	32.44705	34.07995	25.49993	3.58E-06	0.016458	0.785813	Up
ENSCHIG 00000017689	TXNIP	120.1259	167.1237	137.1604	94.74808	75.35614	80.8496	1.17E-05	0.032127	−0.68583	Down
ENSCHIG 00000025767	CSRP2	13.96322	16.28331	12.62957	7.791997	8.4741	8.895063	3.49E-06	0.016458	−0.6999	Down
ENSCHIG 00000021133	THEM5	6.805692	9.054071	5.940543	4.269707	4.11477	2.969	2.67E-05	0.048247	−0.76889	Down

NC, the negative control of SI; FPKM, genes expression levels, cDNA fragments/(mapped fragments×transcript length); SI, the circCOL1A1-si; FDR, false discovery rate, corrected p-value; Log2_FC, the fold change of gene expression.

**Table 2:** The differentially expressed genes in circCOL1A1-overexpressing goat hair follicle stem cells (gHSFCs)

ID	Gene_name	PL2-FPKM	PL3-FPKM	PL4-FPKM	OV2-FPKM	OV3-FPKM	OV4-FPKM	p-value	FDR	Log2_FC	Regulated
ENSCHIG 00000019372	CMTM3	1.621713	1.594154	1.869358	8.683109	9.035161	8.881901	0.000330	0.049800	1.815283	Up
ENSCHIG 00000019848	IFIH1	121.939	118.837	83.96595	44.66761	30.2017	35.9674	9.42E-14	1.85E-10	−1.14303	Down
ENSCHIG 00000017063	IFIT2	155.8014	145.9856	104.762	46.9876	16.78217	19.13442	1.94E-11	1.67E-08	−1.15066	Down
ENSCHIG 00000019892	OAS1	363.0455	506.7122	293.809	140.3063	73.06493	76.47686	7.05E-13	9.67E-10	−1.20726	Down
ENSCHIG 0000002387	EPSTI1	49.98095	96.569	47.59721	18.69568	10.13989	12.38706	6.57E-13	9.67E-10	−1.2266	Down
ENSCHIG 00000025883	DDX58	438.1515	552.5896	352.7583	165.8358	90.2634	105.951	4.41E-14	1.01E-10	−1.23762	Down
ENSCHIG 00000001865	MX1	1186.85	1393.071	882.6482	406.9511	222.0811	215.8587	1.92E-14	5.25E-11	−1.27384	Down
ENSCHIG 00000026535	TRANK1	25.94079	19.34955	16.51923	6.876887	4.816035	6.112134	2.98E-17	4.09E-13	−1.30511	Down
ENSCHIG 00000022954	HERC6	111.3421	142.8068	81.81461	36.08559	21.24255	25.56861	1.24E-15	5.68E-12	−1.31359	Down
ENSCHIG 00000026701	SLFN11	11.20459	20.81793	7.480375	2.410641	1.558708	1.172699	1.32E-14	4.52E-11	−1.31868	Down
ENSCHIG 00000021832	RSAD2	146.2364	135.9759	111.0136	39.79048	16.82507	26.11014	5.65E-16	3.87E-12	−1.35835	Down

PL, the negative control of Over; FPKM, genes expression levels, cDNA fragments/(mapped fragments×transcript length); OV, the circCOL1A1 overexpression; FDR, false discovery rate, corrected p-value; Log2_FC, the fold change of gene expression.

**Table 3:** The differentially expressed miRNAs in circCOL1A1-knockdown goat hair follicle stem cells (gHSFCs) (up Top10 and down Top10)

ID	NC1(TPM)	NC2 (TPM)	NC3 (TPM)	NC4 (TPM)	SI1 (TPM)	SI2 (TPM)	SI3 (TPM)	SI4 (TPM)	p-value	FDR	Log2_FC	Regulated
Chi-miR-483	4.09	22.94	2.08	0.91	122.42	69.67	231.08	69.53	9.41E-05	1.40E-07	2.109249	Up
Chi-miR-1814	0.4	0.39	0.62	0.2	3.84	1.24	5.05	1.22	0.000113	2.00E-06	1.733596	Up
Unconservative_7_12945	3.69	11.41	3.54	2.12	28.26	20.74	47.04	14.88	2.02E-06	1.47E-10	1.698164	Up
Unconservative_6_10953	0.7	3.02	0.42	0.2	5.48	5.07	12.62	3.33	0.000171	3.86E-11	1.696943	Up
Chi-miR-877-3p	0.5	0.26	0.1	0.61	1.85	2.82	3.32	1.44	0.000142	2.23E-05	1.662353	Up
Unconservative_1_415	46.36	76.18	24.96	16.79	222.21	140.02	304.03	120.96	1.37E-06	9.37E-07	1.583659	Up
Chi-let-7b-3p	5.58	4.2	6.34	4.04	21.64	22.77	30.56	15.11	3.86E-11	3.86E-11	1.530748	Up
Unconservative_4_7333	2.19	2.62	1.77	1.92	11.03	7.89	13.16	5.66	9.58E-09	9.58E-09	1.506787	Up
Unconservative_3_4801	273.09	265.89	145.39	86.96	873.82	566.4	1,026.5	480.82	4.69E-06	6.94E-23	1.308046	Up
Chi-miR-134	0.9	0.79	0.52	1.01	4.77	1.47	4.65	2.33	0.001229	0.0012289	1.247493	Up
Chi-miR-451-5p	5.18	8	9.88	9.61	2.21	2.59	2.13	2	7.75E-14	7.87E-06	−2.21047	Down
Chi-miR-19a	42.87	41.96	54.49	57.13	18.51	11.84	16.74	13.66	6.94E-23	1.79E-10	−2.15355	Down
Chi-miR-671-5p	180.86	153.27	181.58	233.27	70.61	71.82	36.81	68.75	9.75E-11	7.75E-14	−1.92998	Down
Chi-miR-15a-5p	2.89	3.8	4.78	2.83	2.35	0	0.66	0.89	2.68E-05	6.94E-23	−1.87728	Down
Chi-miR-19b-3p	1,650.79	1,556.69	2,093.33	1,919.27	877.02	834.16	683.27	769.72	1.08E-13	9.75E-11	−1.63465	Down
Chi-miR-301a-3p	2.19	9.96	1.35	0.71	1.14	0.9	1.59	0.56	0.000707	1.47E-10	−1.62554	Down
Chi-miR-20a-5p	2.19	9.96	1.35	0.71	1.14	0.9	1.59	0.56	0.000707	1.08E-13	−1.62554	Down
Unconservative_2_3134	21.74	17.83	19.66	29.02	11.1	11.84	7.71	9	2.00E-08	1.40E-07	−1.56721	Down
Unconservative_2_3133	4.59	3.02	3.64	2.73	1.42	1.47	2.66	1.33	2.46E-06	1.47E-10	−1.46199	Down
Chi-miR-500-5p	1,454.28	1,675.08	2,280.42	3,005.34	775.03	1,262.35	644.07	1,201.45	2.23E-05	2.00E-08	−1.43635	Down

NC, the negative control of SI; TPM, miRNAs expression levels, the read-counts×1000000/mapped-reads; SI, the circCOL1A1-si; FDR, false discovery rate, corrected p-value; Log2_FC, the fold change of miRNA expression.

**Table 4:** The differentially expressed miRNAs in circCOL1A1-overexpressing goat hair follicle stem cells (gHSFCs) (up Top10 and down Top10)

ID	PL1 (TPM)	PL2 (TPM)	PL3 (TPM)	PL4 (TPM)	OV1 (TPM)	OV2 (TPM)	OV3 (TPM)	OV4 (TPM)	p-value	FDR	Log2_FC	Regulated
Chi-mir-451-5p	3.6	1.63	1.57	1.39	7.57	4.51	6.03	29.36	0.000143	0.000143	1.804477	Up
Chi-mir-221-5p	74.4	36.62	37.51	38.1	153	136.39	90.31	87.12	7.46E-11	2.00E-06	1.476446	Up
Chi-mir-30a-5p	68.24	81.49	81.31	99.51	175.39	153.58	188.96	160.09	1.90E-13	1.90E-13	1.32519	Up
Unconservative_2_3133	0.34	0.7	0.79	0.09	1.49	0.28	2.32	1.16	0.002985	3.45E-08	1.322843	Up
Unconservative_2_3134	0.34	0.7	0.79	0.09	1.49	0.28	2.32	1.16	0.002985	4.45E-06	1.322843	Up
Chi-mir-126-3p	70.12	77.18	97.42	63.9	128.27	115.73	155.39	217.74	2.24E-10	2.24E-10	1.30876	Up
Chi-mir-671-5p	1.54	1.16	1.47	2.03	3.95	3.38	3.15	2.85	1.36E-05	3.03E-12	1.283527	Up
Chi-mir-19a	13.01	8.49	7.07	7.68	23.99	18.22	14.46	14.36	1.28E-05	1.28E-05	1.189487	Up
Chi-mir-7-5p	1,551.4	799.63	887.94	878.27	2542.1	2,192.22	1,657.87	1,564.44	2.30E-05	2.98E-10	1.163996	Up
Chi-mir-146b-5p	9.42	6.04	9.82	7.4	14.39	10.9	10.38	20.27	0.000121	0.000121	1.031556	Up
Chi-mir-877-3p	1.46	3.6	2.65	1.85	0.43	0.19	0.37	0.32	6.55E-07	3.03E-12	−1.96073	Down
Chi-mir-128-3p	53.08	74.63	89.56	22.84	12.26	7.51	14.28	9.61	3.01E-11	3.01E-11	−1.87094	Down
Chi-mir-27a-3p	4,657.2	6,546.28	9,323.4	3,274.57	1,247.7	948.73	1,352.46	1,119.98	4.30E-18	4.30E-18	−1.8502	Down
Chi-let-7b-3p	11.56	17.67	7.56	14.33	3.31	1.6	3.99	2.01	2.96E-08	2.96E-08	−1.69913	Down
Chi-let-7d-3p	30.14	35.22	33.68	20.9	7.89	5.92	7.05	8.03	2.76E-19	2.76E-19	−1.62503	Down
Chi-mir-149-5p	52.48	78.35	104.88	34.4	16.85	10.43	16.41	17.85	7.46E-11	7.46E-11	−1.60932	Down
Unconservative_5_8882	110.79	178.2	178.14	110.42	43.5	24.7	39.68	39.49	1.22E-17	1.22E-17	−1.53444	Down
Unconservative_3_5273	1,052.59	1,397.23	1,082.19	541	360.07	147.48	252.47	310.04	5.84E-08	4.49E-11	−1.42653	Down
Chi-mir-3959-3p	3.6	5.11	6.87	3.14	0.85	1.5	1.21	2.01	6.83E-05	6.83E-05	−1.20681	Down
Chi-mir-151-3p	3,563.77	4,874.72	4,906.18	5,092.32	1,205.27	1,670.23	1,847.01	1,550.93	2.98E-10	2.98E-10	−1.15791	Down

PL, the negative control of Over; TPM: miRNAs expression levels, the read-counts×1000000/mapped-reads; OV, the circCOL1A1 overexpression; FDR, false discovery rate, corrected p-value; Log2_FC, the fold change of miRNA expression.

**Table 5:** The differentially expressed circRNAs in circCOL1A1-knockdown goat hair follicle stem cells (gHSFCs) (up Top10 and down Top10)

ID	NC1 (TPM)	NC2 (TPM)	NC3 (TPM)	NC4 (TPM)	SI1 (TPM)	SI2 (TPM)	SI3 (TPM)	SI4 (TPM)	Source	FDR	Log2_FC	Regulated
10:22433524|22443222	0	38.09814	0	0	1,044.659	385.9514	1,097.351	86.18461	*ACTN1*	5.28E-05	3.232885	UP
19:53919988|53921443	0	0	0	0	522.3296	128.6505	862.2041	287.282	*SEC14L1*	0.000157	3.112552	UP
4:19492379|19496495	0	0	0	0	326.456	300.1844	235.1466	632.0205	*PTN*	0.000384	2.938191	UP
18:44114399|44115249	0	0	0	0	65.2912	300.1844	235.1466	430.923	*DPY19L3*	0.004703	2.357539	UP
4:32171093|32179007	0	152.3926	0	0	457.0384	986.3202	235.1466	459.6512	*WASL*	0.00315	2.34827	UP
21:41031868|41043602	0	0	0	207.3583	522.3296	471.7183	940.5863	459.6512	*STRN3*	0.005207	2.182736	UP
17:3756437|3773508	0	38.09814	0	0	783.4944	128.6505	313.5288	28.7282	*PITPNB*	0.010272	2.138703	UP
7:104399169|104402324	0	0	0	0	130.5824	257.3009	313.5288	143.641	*UPF1*	0.010466	2.129589	UP
29:41217172|41224401	61.44393	0	0	0	718.2032	300.1844	235.1466	28.7282	*AHNAK*	0.010567	2.127143	UP
6:63864968|63873468	0	0	0	0	261.1648	128.6505	313.5288	86.18461	*GUF1*	0.01572	2.002356	UP
16:78675219|78678516	614.4393	380.9814	352.6626	59.24522	0	0	0	0	*PPP1R12B*	0.002289	−2.54084	DOWN
19:17779051|17786230	245.7757	190.4907	503.8037	236.9809	0	0	0	0	*CRLF3*	0.004093	−2.39427	DOWN
19:10901081|10912990	675.8833	304.7851	1007.607	236.9809	0	85.76697	0	57.4564	*BRIP1*	0.002895	−2.32726	DOWN
24:2399338|2411998	368.6636	1,409.631	453.4233	236.9809	0	214.4174	78.38219	0	*ZNF236*	0.012061	−1.9612	DOWN
2:92159817|92165129	430.1075	495.2758	1,158.749	385.0939	0	171.5339	156.7644	0	*STAM2*	0.011346	−1.93639	DOWN
3:70329789|70331805	61.44393	495.2758	755.7056	473.9617	0	128.6505	0	57.4564	*CCDC18*	0.019726	−1.86885	DOWN
3:42540162|42557095	307.2197	76.19628	503.8037	385.0939	0	85.76697	0	0	*MIER1*	0.026850	−1.83679	DOWN
14:65554072|65565953	491.5515	190.4907	554.1841	0	0	42.88349	0	0	*ATAD2*	0.029157	−1.81866	DOWN
6:116844051|116875399	245.7757	419.0795	0	651.6974	0	85.76697	0	0	*novel gene*	0.032595	−1.77769	DOWN
24:61335363|61361504	245.7757	342.8833	0	503.5843	0	42.88349	0	28.7282	*PHLPP1*	0.037489	−1.73451	DOWN

NC, the negative control of SI; TPM, circRNAs expression levels, the read-counts×1000000/mapped-reads; SI, the circCOL1A1-si; FDR, false discovery rate, corrected p-value; Log2_FC, the fold change of circRNAs expression.

**Table 6:** The differentially expressed circRNAs in circCOL1A1-overexpressing goat hair follicle stem cells (gHSFCs) (up Top10 and down Top10)

ID	PL1 (TPM)	PL2 (TPM)	PL3 (TPM)	PL4 (TPM)	OV1 (TPM)	OV2 (TPM)	OV3 (TPM)	OV4 (TPM)	Source	FDR	Log2_FC	Regulated
19:36112781|36113653	0	0	0	0	1,1940.3	1,7327.41	6,306.164	1,0111.3	*COL1A1*	3.45E-31	7.860259	UP
6:38004821|38018156	0	0	0	0	318.408	548.3358	39.41353	800.1746	*LCORL*	0.000376	3.131878	UP
1:136780642|136784461	0	0	0	0	79.60199	219.3343	512.3758	290.9726	*DNAJC13*	0.002992	2.643697	UP
10:56418459|56419642	0	0	0	0	517.4129	109.6672	197.0676	218.2294	*CA12*	0.003951	2.568923	UP
12:50865978|50873192	0	66.19886	97.72305	0	716.4179	438.6686	275.8947	581.9452	*IFT88*	0.002691	2.455704	UP
25:13376850|13386108	0	0	0	49.70426	278.607	109.6672	591.2029	290.9726	*PARN*	0.005486	2.439790	UP
11:87695950|87701722	0	66.19886	0	0	0	219.3343	394.1353	1,091.147	*ASAP2*	0.005731	2.437655	UP
17:13083954|13146806	0	0	0	99.40852	437.8109	164.5007	315.3082	727.4314	*FBXW8*	0.004733	2.425539	UP
6:67622313|67629625	0	0	0	0	39.801	438.6686	236.4812	218.2294	*SLAIN2*	0.007479	2.386417	UP
17:9318639|9329329	0	0	0	49.70426	318.408	54.83358	591.2029	218.2294	*PTPN11*	0.009515	2.293250	UP
2:104706111|104715418	333.3175	529.5909	228.0205	347.9298	0	0	0	0	*COBLL1*	0.001324	−2.84128	DOWN
15:29829882|29851190	714.2517	198.5966	162.8718	447.3383	0	0	0	0	*RAB6A*	0.001586	−2.79954	DOWN
3:15674605|15678736	666.6349	264.7954	358.3179	49.70426	0	0	0	0	*SCMH1*	0.004259	−2.54695	DOWN
17:63346672|63473483	999.9524	132.3977	130.2974	149.1128	0	0	0	0	*LRBA*	0.004837	−2.51191	DOWN
9:9360166|9382937	1,047.569	66.19886	130.2974	149.1128	0	0	0	0	*IBTK*	0.006464	−2.42938	DOWN
2:135477860|135480092	1,809.438	66.19886	130.2974	49.70426	0	0	39.41353	0	*CYFIP1*	0.007044	−2.38777	DOWN
10:48825620|48829253	571.4014	66.19886	228.0205	198.817	0	0	0	0	*RXF7*	0.011206	−2.26238	DOWN
14:91213722|91243348	0	397.1932	716.6357	248.5213	0	54.83358	0	0	*FAM135A*	0.016381	−2.13376	DOWN
26:29240204|29244032	714.2517	463.392	553.764	198.817	0	164.5007	0	0	*FBXW4*	0.014464	−2.08224	DOWN
1:134679232|134731778	428.551	66.19886	293.1692	99.40852	0	0	0	0	*RYK*	0.02146	−2.04515	DOWN

PL, the negative control of Over; TPM, circRNAs expression levels, the read-counts×1000000/mapped-reads; OV, the circCOL1A1 overexpression; FDR, false discovery rate, corrected p-value; Log2_FC, the fold change of circRNAs expression.

## SUPPLEMENTARY MATERIAL

**Supplement 1**. Primer sequence information for differentially expressed miRNAs

**Supplement 2**. Primer sequence information for differentially expressed genes

**Supplement 3**. Primer sequence information for differentially expressed circRNAs

**Supplement 4**. The clean data of different samples of genes and circRNAs part

**Supplement 5**. The clean data of different samples of miRNAs part

**Supplement 6**. The mapped reads of different samples of genes part

**Supplement 7**. The mapped reads of different samples of circRNAs part

**Supplement 8**. The mapped reads of different samples of miRNAs part (collapsed)

**Supplements 9**. Characteristics of genes related to circCOL1A1-overexpressing and circCOL1A1-knockdown goat hair follicle stem cells (gHFSCs).

**Supplements 10**. Characteristics of miRNAs in circCOL1A1-overexpressing and circCOL1A1-knockdown goat hair follicle stem cells (gHFSCs).

**Supplements 11**. Characteristics of circRNAs in circCOL1A1-overexpressing and circCOL1A1-knockdown goat hair follicle stem cells (gHFSCs).
