# Supplementary material for: Integrative and comparative analysis of whole-transcriptome sequencing in circCOL1A1-knockdown and circCOL1A1-overexpressing goat hair follicle stem cells
Source: Anim Biosci. 2025 Feb 27;38(6):1116–39. doi: 10.5713/ab.24.0816 (PMC12061571; doi:10.5713/ab.24.0816)
Supplement: Supplementary file 1 [file ab-24-0816-Supplementary-1.pdf]

Supplement 1. Primer sequence information for differentially expressed miRNAs

Name	Sequence Name	Sequence Information (5' to 3')
18S-rRNA	18S-rRNA-F	GTGGTGTTGAGGAAAGCAGACA
ID: 493779	18S-rRNA-R	TGATCACACGTTCCACCTCATC
Chi-miR-483	miR-483-F	CACTCCTCTCCTCCCGT
	miR-483-R	GTGCAGGGTCCGAGGT
Chi-miR-1814	miR-1814-F	GTTTTGTTTGGGTTTGT
	miR-1814-R	GTGCAGGGTCCGAGGT
Chi-miR-877-3p	miR-877-3p-F	TCCTCTTCTCCCTCCTC
	miR-877-3p-R	GTGCAGGGTCCGAGGT
Chi-let-7b-3p	let-7b-3p-F	CTATACAACCTACTGCC
	let-7b-3p-R	GTGCAGGGTCCGAGGT
Chi-miR-145-5p	miR-145-5p-F	GTCCAGTTTTCCCAGGA
	miR-145-5p-R	GTGCAGGGTCCGAGGT
Chi-miR-29b-3p	miR-29b-3p-F	TAGCACCATTTGAAATC
	miR-29b-3p-R	GTGCAGGGTCCGAGGT
Chi-miR-17-5p	miR-17-5p-F	CAAAGTGCTTACAGTGC
	miR-17-5p-R	GTGCAGGGTCCGAGGT
Chi-miR-7-5p	miR-7-5p-F	TGGAAGACTAGTGATTTT
	miR-7-5p-R	GTGCAGGGTCCGAGGT
Chi-miR-19b-3p	miR-19b-3p-F	TGTGCAAATCCATGCAA
	miR-19b-3p-R	GTGCAGGGTCCGAGGT

Chi-miR-671-5p	miR-671-5p-F	AGGAAGCCCTGGAGGGGCT
	miR-671-5p-R	GTGCAGGGTCCGAGGT
Chi-miR-451-5p	miR-451-5p-F	AAACCGTTACCATTACT
	miR-451-5p-R	GTGCAGGGTCCGAGGT
Chi-miR-221-5p	miR-221-5p-F	ACCTGGCATACAATGTA
	miR-221-5p-R	GTGCAGGGTCCGAGGT
Chi-miR-30a-5p	miR-30a-5p-F	TGTAAACATCCTCGACTG
	miR-30a-5p-R	GTGCAGGGTCCGAGGT
Chi-miR-146b-5p	miR-146b-5p-F	TGAGAACTGAATTCCATA
	miR-146b-5p-R	GTGCAGGGTCCGAGGT
Chi-miR-151-3p	miR-151-3p-F	CTAGACTGAAGCTCCTT
	miR-151-3p-R	GTGCAGGGTCCGAGGT
Chi-miR-149-5p	miR-149-5p-F	TCTGGCTCCGTGTCTTC
	miR-149-5p-R	GTGCAGGGTCCGAGGT
Chi-miR-27a-3p	miR-27a-3p-F	TTCACAGTGGCTAAGTT
	miR-27a-3p-R	GTGCAGGGTCCGAGGT
Chi-miR-128-3p	miR-128-3p-F	TCACAGTGAACCGGTCT
	miR-128-3p-R	GTGCAGGGTCCGAGGT
