# Supplementary material for: Integrative and comparative analysis of whole-transcriptome sequencing in circCOL1A1-knockdown and circCOL1A1-overexpressing goat hair follicle stem cells
Source: Anim Biosci. 2025 Feb 27;38(6):1116–39. doi: 10.5713/ab.24.0816 (PMC12061571; doi:10.5713/ab.24.0816)
Supplement: Supplementary file 2 [file ab-24-0816-Supplementary-2.pdf]

Supplement 2. Primer sequence information for differentially expressed genes

Name	Sequence Name	Sequence Information (5' to 3')
GAPDH	GAPDH-F	AGGTCGGAGTGAACGGATTC
ID:100860872	GAPDH-R	CCAGCATCACCCCACTTGAT
MYO16	MYO16-F	CGATGTCAACCACCAGGAT
ID: 102170796	MYO16-R	CCATACTTTGCTGCCAGGT
HAPLN4	HAPLN4-F	GTCCTGAGAGTGACCCAACG
ID: 106502262	HAPLN4-R	AGCCCTATGGAGCAAGTTCG
TBKBP1	TBKBP1-F	GCCTCCCACTTTGCCCTGAT
ID: 102172659	TBKBP1-R	GCTGTTCTTCCTGCTCCTTGT
CPXM1	CPXM1-F	TCCTGACGGCTATGAGACTGC
ID: 102186410	CPXM1-R	TGGAGGTTGGCACTTAGCAC
TXNIP	TXNIP-F	TCGTTTTCTGAGGTGGGACG
ID: 102183379	TXNIP-R	GTACACCTTTTCGGGGTCGT
CSRP2	CSRP2-F	CAAGGGATTTGGCTACGGC
ID: 102181652	CSRP2-R	TGCTCGGTTCTGGGTTTGC
THEM5	THEM5-F	CAGGCACTTTCCTCCAGAT
ID: 102190478	THEM5-R	CCTCACTTCACCCGCTACT
CMTM3	CMTM3-F	CCTCTGCTTCCTCTTTGCTGATG
ID: 102174055	CMTM3-R	ACGGCTGTGATGGAGATGGC
MX1	MX1-F	AGTCCTCCGACTCTTCACTCA
ID: 102188635	MX1-R	CCAGCACAGTTCCTGACACA

SAMD9	SAMD9-F	CGGCACACCCATACTGAGAA
ID: 102180452	SAMD9-R	TGCCATTCTGATACCCCGATT
GBP5	GBP5-F	AAAAGGAGATCGAAGGTATAACAGA
ID: 102174264	GBP5-R	CTCTGCTCCATCATTTGCTGAT
STAT1	STAT1-F	CTTCGCTGAGTTAGCCGACA
ID: 102189170	STAT1-R	TACCACTGGGACATCTCTGCG
STAT2	STAT2-F	CCCCTGTGGAAGTGACGAG
ID: 102190301	STAT2-R	GCCTGAATGTCCCGATAGAA
EPSTI1	EPSTI1-F	CGAAGAAATGAGATACAACAGA
ID: 102177448	EPSTI1-R	ATGGGCGGTAGGGTAATGG
RNF213	RNF213-F	GCTTTCCAGTTGTGTCGCTG
ID: 102174427	RNF213-R	TTCCTTCTAAGCACCCACCG
PARP9	PARP9-F	AGTTTCAGTTTCGCTTTCCCG
ID: 102187626	PARP9-R	CTGTATTCCCCTTTCTCCACTGA
