# Supplementary material for: Integrative and comparative analysis of whole-transcriptome sequencing in circCOL1A1-knockdown and circCOL1A1-overexpressing goat hair follicle stem cells
Source: Anim Biosci. 2025 Feb 27;38(6):1116–39. doi: 10.5713/ab.24.0816 (PMC12061571; doi:10.5713/ab.24.0816)
Supplement: Supplementary file 3 [file ab-24-0816-Supplementary-3.pdf]

Supplement 3. Primer sequence information for differentially expressed circRNAs

Name	Sequence Name	Sequence Information (5' to 3')
GAPDH	GAPDH-F	AGGTCGGAGTGAACGGATTC
ID:100860872	GAPDH-R	CCAGCATCACCCCACTTGAT
10:22433524|22443222	circACTN1-F	AAGGGCTTCTCTTGTGGTGT
circACTN1	circACTN1-R	CCCTACTTCCAAATGTGCCC
15:36663076|36665327	circZNF215-F	AGACTACTACTTTGGGATTG
circZNF215	circZNF215-R	TACAATCTTGAGGGCATAG
21:67540278|67542568	circKLC1-F	GTCTGTGCCAGGAGAACCA
circKLC1	circKLC1-R	TTGATTTCTCCTCCACCAG
21:41031868|41043602	circSTRN3-F	TGACCTTTTCTTTCTCCTT
circSTRN3	circSTRN3-R	ATCCTGAATGGAGGTGAAT
21:1465717|1525199	circUBE3A-F	AATGGCAGTCATCCAAATC
circUBE3A	circUBE3A-R	TACAACAGGCACAGACAGG
10:19974380|19976180	circSIPA1L1-F	GGGACTCTGTGACTGCCATAAT
circSIPA1L1	circSIPA1L1-R	CTGGTGATGAGCTGTCCGTA
2:112169304|112176967	circITGA6-F	GGAGTTCCCTGGTAGTGTT
circITGA6	circITGA6-R	TGTGACGGTTTGCTGAATA
16:78675219|78678516	circPPP1R12B-F	GATGCTCTACCCTCGACCG
circPPP1R12B	circPPP1R12B-R	AGGAAGATGAAACGGAAGTCCC
24:2399338|2411998	circZNF236-F	GGCGTTGAAACTGGGACTCTT
circZNF236	circZNF236-R	TCACCCCTCCTAACCTCCTA

19:17779051|17786230	circCRLF3-F	AGCAGTCGGAGAAACATAGC
circCRLF3	circCRLF3-R	TCACCAATCGCTCATCCAG
19:36112781|36113653	circCOL1A1-F	TATCCTCCACTCCACTCACA
circCOL1A1	circCOL1A1-R	CTTGAATACCAGTGGGACCAG
17:18339237|18340825	circKNTC1-F	GGTGCTCCCTGTCATCTAT
circKNTC1	circKNTC1-R	CTGCCCAGTGTCGGTATTG
1:50172587|50186023	circCBLB-F	TCATCGTCATCTTCCTCTACCG
circCBLB	circCBLB-R	CTTCAGCCAGTTCGCAGAGATA
12:84198435|84203775	circDIAPH3-F	ATAATCCAAGCCCCTCACG
circDIAPH3	circDIAPH3-R	TTGGGTTATTTGCTGTTTG
6:38004821|38018156	circLCORL-F	AGCCGTGGTCCATAAAGCC
circLCORL	circLCORL-R	ATTGAATGCCAAGCAGAAA
1:132757978|132765282	circPPP2R3A-F	TCCACAGAAACACTCACCCTG
circPPP2R3A	circPPP2R3A-R	TTTCCGGCATTCTGTGGTGT
8:76826912|76847452	circGKAP1-F	AACCACTGCCACTATCCAC
circGKAP1	circGKAP1-R	TTGATTCAGGGATGTAGGC
16:22769716|22782249	circMARC2-F	ACAAGTCGTCCCCACTGTTTG
circMARC2	circMARC2-R	GAAGGAAGCCGGAAGGGAAGG
1:134679232|134731778	circRYK-F	GATGGCTCTTGAAAGTCTGG
circRYK	circRYK-R	CACCCACTGACCCTCTTGT
2:104706111|104715418	circCOBLL1-F	CTCCACCTCCAACATTCCT
circCOBLL1	circCOBLL1-R	TCTTGTATGGTGAAATCCC

10:48825620|48829253	circRFX7-F	CACAAGACGGCAGGCAGAA
circRFX7	circRFX7-R	CTATCGGTATCGTCGTGGC
