# Supplementary material for: Integrative and comparative analysis of whole-transcriptome sequencing in circCOL1A1-knockdown and circCOL1A1-overexpressing goat hair follicle stem cells
Source: Anim Biosci. 2025 Feb 27;38(6):1116–39. doi: 10.5713/ab.24.0816 (PMC12061571; doi:10.5713/ab.24.0816)
Supplement: Supplementary file 4 [file ab-24-0816-Supplementary-4.pdf]

Supplemental Files

Supplement 4. The clean data of different samples of genes and circRNAs part

Sample	ReadSum	BaseSum	GC (%)	Q20 (%)	Q30 (%)
NC-1	51,155,983	15,346,794,900	42.96%	98.42%	94.86%
NC-2	47,516,484	14,254,945,200	44.23%	98.47%	94.89%
NC-3	57,515,802	17,254,740,600	43.30%	98.51%	95.09%
NC-4	57,676,821	17,303,046,300	44.54%	98.56%	95.24%
SI-1	52,073,874	15,622,162,200	44.23%	98.46%	94.91%
SI-2	62,748,541	18,824,562,300	44.64%	98.57%	95.30%
SI-3	49,726,511	14,917,953,300	42.70%	98.48%	95.06%
SI-4	55,121,112	16,536,333,600	44.47%	98.56%	95.28%
Plc5-1	59,644,942	17,893,482,600	44.07%	98.31%	94.76%
Plc5-2	39,766,169	11,929,850,700	43.74%	98.58%	95.31%
Plc5-3	56,236,780	16,871,034,000	44.28%	98.61%	95.36%
Plc5-4	42,759,080	12,827,724,000	43.84%	98.49%	95.04%
Over-1	53,422,055	16,026,616,500	44.41%	98.61%	95.40%
Over-2	34,181,938	10,254,581,400	44.62%	98.52%	95.20%
Over-3	59,315,492	17,794,647,600	44.00%	98.65%	95.56%
Over-4	38,959,089	11,687,726,700	42.91%	98.59%	95.39%

Note: Sample: the name of Sample (NC: the negative control of SI, SI: the circCOL1A1-si, Plc5: the

negative control of Over, Over: the circCOL1A1 overexpression); ReadSum: the sum total of pair-end reads; BaseSum: the sum total of bases; GC: the percent of G and C bases; Q20: the percent of bases quality ≥ 20 in total; Q30: the percent of bases quality ≥ 30 in total.
