# Supplementary material for: Integrative and comparative analysis of whole-transcriptome sequencing in circCOL1A1-knockdown and circCOL1A1-overexpressing goat hair follicle stem cells
Source: Anim Biosci. 2025 Feb 27;38(6):1116–39. doi: 10.5713/ab.24.0816 (PMC12061571; doi:10.5713/ab.24.0816)
Supplement: Supplementary file 5 [file ab-24-0816-Supplementary-5.pdf]

Supplement 5. The clean data of different samples of miRNAs part

Sample	Raw-reads	Raw Base(bp)	Clean reads	Clean Base(bp)	Q20	Q30	GC
NC-1	12,121,848	606,092,400	12,121,841	606,092,050	99.29	97.41	46.96
NC-2	10,254,798	512,739,900	10,254,795	512,739,750	99.40	98.00	48.09
NC-3	11,983,481	599,174,050	11,983,026	599,151,300	99.27	97.84	47.04
NC-4	12,799,479	639,973,950	12,799,469	639,973,450	99.32	97.56	46.94
SI-1	19,732,821	986,641,050	19,732,818	986,640,900	99.45	98.19	48.98
SI-2	11,551,396	577,569,800	11,551,395	577,569,750	99.45	97.96	48.30
SI-3	13,319,584	665,979,200	13,318,160	665,907,980	99.38	98.09	50.92
SI-4	11,913,037	595,651,850	11,911,814	595,590,650	99.43	98.26	48.07
Plc5-1	13,983,084	699,154,200	13,982,543	699,126,759	99.35	97.64	47.18
Plc5-2	12,105,537	605,276,850	12,104,270	605,213,445	99.35	97.60	48.86
Plc5-3	13,993,521	699,676,050	13,993,518	699,675,900	99.47	98.22	48.41
Plc5-4	13,009,070	650,453,500	13,009,067	650,453,318	99.48	98.28	47.26
Over-1	11,347,308	567,365,400	11,347,170	567,358,476	99.34	98.00	46.82
Over-2	12,596,257	629,812,850	12,596,252	629,812,586	99.46	98.26	46.60
Over-3	12,763,416	638,170,800	12,763,247	638,162,340	99.38	98.02	46.90
Over-4	11,545,530	577,276,500	11,545,522	577,276,086	99.42	97.97	46.94

Note: Sample: the name of Sample (NC: the negative control of SI, SI: the circCOL1A1-si, Plc5: the negative control of Over, Over: the circCOL1A1 overexpression); Raw reads: the sum total of

single-end reads; Raw Base(bp): the sum total of bases in Raw Data; Clean-reads: the sum total of pair-end reads; Clean Base(up): the sum total of bases in Clean Data; Q20: the percent of bases quality ≥ 20 in total; Q30: the percent of bases quality ≥ 30 in total; GC: the percent of G and C bases in Clean Data.
