# Supplementary material for: Integrative and comparative analysis of whole-transcriptome sequencing in circCOL1A1-knockdown and circCOL1A1-overexpressing goat hair follicle stem cells
Source: Anim Biosci. 2025 Feb 27;38(6):1116–39. doi: 10.5713/ab.24.0816 (PMC12061571; doi:10.5713/ab.24.0816)
Supplement: Supplementary file 6 [file ab-24-0816-Supplementary-6.pdf]

Supplement 6. The mapped reads of different samples of genes part

Sample	Total Reads	Reads mapped
NC-1	51155983	97.73%
NC-2	47516484	97.86%
NC-3	57515802	97.27%
NC-4	57676821	97.90%
SI-1	52073874	97.37%
SI-2	62748541	90.66%
SI-3	49726511	97.49%
SI-4	55121112	97.84%
Plc5-1	59644942	97.07%
Plc5-2	39766169	97.69%
Plc5-3	56236780	97.71%
Plc5-4	42759080	97.73%
Over-1	53422055	97.61%
Over-2	34181938	97.69%
Over-3	59315492	97.82%
Over-4	38959089	97.79%

Note: Sample: the name of Sample (NC: the negative control of SI, SI: the circCOL1A1-si, Plc5: the negative control of Over, Over: the circCOL1A1 overexpression); Total reads: the number of clean reads; Reads mapped: total mapped reads in genome.
