# Supplementary material for: Integrative and comparative analysis of whole-transcriptome sequencing in circCOL1A1-knockdown and circCOL1A1-overexpressing goat hair follicle stem cells
Source: Anim Biosci. 2025 Feb 27;38(6):1116–39. doi: 10.5713/ab.24.0816 (PMC12061571; doi:10.5713/ab.24.0816)
Supplement: Supplementary file 7 [file ab-24-0816-Supplementary-7.pdf]

Supplement 7. The mapped reads of different samples of circRNAs part

Sample	Total read	Reads mapped	Multi mapped	Uniq mapped	Unmapped
NC-1	102311966	102259903(99.95%)	20657171(20.19%)	81602732(79.76%)	52063(0.05%)
NC-2	95032968	94965037(99.93%)	22828485(24.02%)	72136552(75.91%)	67931(0.07%)
NC-3	115031604	114761340(99.77%)	23171710(20.14%)	91589630(79.62%)	270264(0.23%)
NC-4	115353642	115263290(99.92%)	28458611(24.67%)	86804679(75.25%)	90352(0.08%)
SI-1	104147748	103946751(99.81%)	22090805(21.21%)	81855946(78.60%)	200997(0.19%)
SI-2	125497082	122497371(97.61%)	30194228(24.06%)	92303143(73.55%)	2999711(2.39%)
SI-3	99453022	99382798(99.93%)	18979773(19.08%)	80403025(80.85%)	70224(0.07%)
SI-4	110242224	110166140(99.93%)	27831718(25.25%)	82334422(74.69%)	76084(0.07%)
Plc5-1	119289884	119152525(99.88%)	26018351(21.81%)	93134174(78.07%)	137359(0.12%)
Plc5-2	79532338	79462712(99.91%)	17956868(22.58%)	61505844(77.33%)	69626(0.09%)
Plc5-3	112473560	112327166(99.87%)	26973425(23.98%)	85353741(75.89%)	146394(0.13%)
Plc5-4	85518160	85455674(99.93%)	19519080(22.82%)	65936594(77.10%)	62486(0.07%)
Over-1	106844110	106684055(99.85%)	25516809(23.88%)	81167246(75.97%)	160055(0.15%)
Over-2	68363876	68308586(99.92%)	16623194(24.32%)	51685392(75.60%)	55290(0.08%)
Over-3	118630984	118557791(99.94%)	27246317(22.97%)	91311474(76.97%)	73193(0.06%)
Over-4	77918178	77868932(99.94%)	15257161(19.58%)	62611771(80.36%)	49246(0.06%)

Note: Sample: the name of Sample (NC: the negative control of SI, SI: the circCOL1A1-si, Plc5: the negative control of Over, Over: the circCOL1A1 overexpression); Total reads: the number of clean

reads; Reads mapped: total mapped reads in genome; Multi mapped: multiple mapped reads in genome; Uniq-mapped: unique mapped reads in genome; Unmapped: none mapped reads in genome.
