# Supplementary material for: Integrative and comparative analysis of whole-transcriptome sequencing in circCOL1A1-knockdown and circCOL1A1-overexpressing goat hair follicle stem cells
Source: Anim Biosci. 2025 Feb 27;38(6):1116–39. doi: 10.5713/ab.24.0816 (PMC12061571; doi:10.5713/ab.24.0816)
Supplement: Supplementary file 8 [file ab-24-0816-Supplementary-8.pdf]

Supplement 8. The mapped reads of different samples of miRNAs part (collapsed)

Sample	Total reads	Total mapped reads	miRNA	exon	intron	scRNA	rRNA	tRNA	snRNA	snoRNA	ncRNA	Pseu
NC-1	135638	94410(69.60%)	3008/1673	7614/6984	9344/9504	0/0	357/61	213/111	323/389	2590/3683	1002/1127	35/76
NC-2	139156	98243(70.60%)	2999/1639	7080/6236	9631/9805	0/0	331/90	229/105	240/290	2138/3047	1002/1171	31/57
NC-3	143359	95302(66.48%)	3495/1893	7254/6654	9557/9810	0/0	301/59	246/127	353/503	2584/3688	856/1090	39/69
NC-4	199199	136840(68.70%)	2994/1636	11545/11014	15318/15911	0/0	497/77	252/136	421/506	3993/5422	1194/1462	63/102
SI-1	179041	137776(76.95%)	4343/2371	11611/10950	13883/14222	0/0	327/59	248/116	407/472	2435/3376	1393/1634	68/95
SI-2	119203	86391(72.47%)	3184/1795	5830/5560	8312/8436	0/0	227/52	173/103	262/298	1756/2400	880/1013	30/55
SI-3	146719	109241(74.46%)	3382/1849	8329/8172	9881/10256	0/0	253/76	188/96	277/369	1812/2539	1075/1259	44/64
SI-4	139975	100660(71.91%)	3109/1735	6929/6374	10412/10474	0/0	267/50	171/87	195/259	1993/2764	925/1193	40/58
Plc5-1	182350	128533(70.49%)	3813/2080	11915/11659	12842/12937	0/0	380/63	209/119	335/450	2936/3955	1141/1386	62/115
Plc5-2	137214	101231(73.78%)	3416/1872	8054/7730	9894/9909	0/0	257/62	198/81	311/346	2009/2625	1005/1117	50/79
Plc5-3	174430	133484(76.53%)	3806/2089	12096/11598	13599/13407	0/0	329/75	202/126	281/368	2495/3236	1298/1468	64/128

Plc5-4	135885	103256(75.99%)	3459/1880	9184/9065	10750/10832	0/0	279/61	159/83	291/327	2564/3376	895/1095	59/91
Over-1	155682	112895(72.52%)	3298/1841	9965/8929	11738/12349	0/0	416/56	240/144	359/458	3094/4333	1050/1447	59/88
Over-2	122976	88311(71.81%)	3084/1708	6323/5730	9823/9857	0/0	342/39	200/108	289/332	2714/3773	823/1041	32/57
Over-3	125997	91302(72.46%)	3590/2031	7144/6609	9338/9614	0/0	294/41	186/91	280/356	2383/3420	863/1115	41/59
Over-4	125820	89209(70.90%)	2979/1696	7404/6836	9365/9389	0/0	302/53	187/102	322/311	2648/3690	781/1056	32/57

Note: Sample: the name of Sample (NC: the negative control of SI, SI: the circCOL1A1-si, Plc5: the negative control of Over, Over: the circCOL1A1 overexpression); Total reads: the number of clean reads; Total mapped reads: total mapped reads in genome; miRNA: the number of microRNA; exon: mapped reads in exon; intron: mapped reads in intron; scRNA: mapped reads in scRNA; rRNA: mapped reads in rRNA; tRNA: mapped reads in tRNA; snRNA: mapped reads in snRNA; snoRNA: mapped reads in snoRNA; ncRNA: mapped reads in ncRNA; Pseu: mapped reads in pseudogene.
