# Supplementary material for: Integrative and comparative analysis of whole-transcriptome sequencing in circCOL1A1-knockdown and circCOL1A1-overexpressing goat hair follicle stem cells
Source: Anim Biosci. 2025 Feb 27;38(6):1116–39. doi: 10.5713/ab.24.0816 (PMC12061571; doi:10.5713/ab.24.0816)
Supplement: Supplementary file 9 [file ab-24-0816-Supplementary-9.pdf]

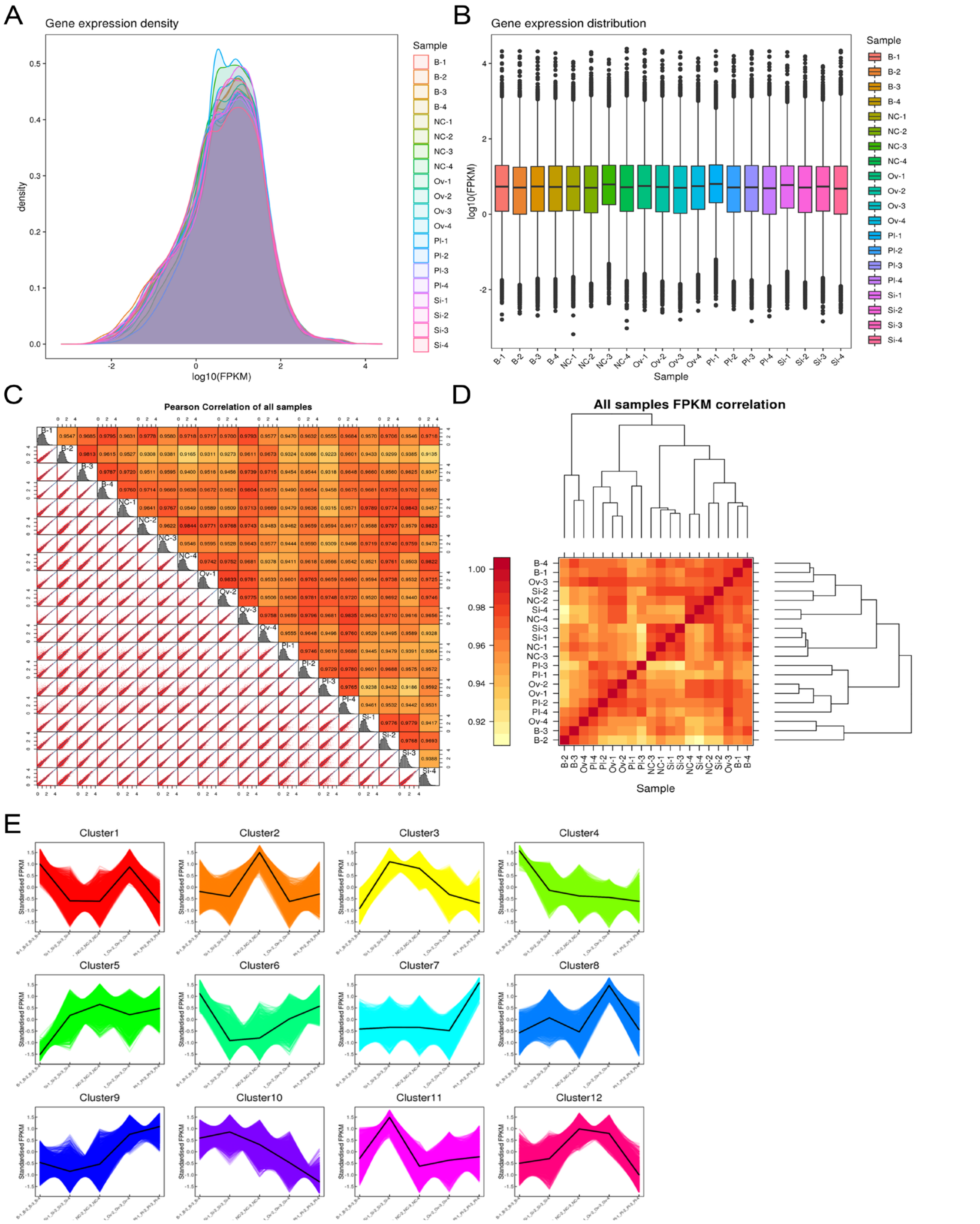

Supplement 9. Characteristics of genes related to circCOL1A1-overexpressing and circCOL1A1-knockdown goat hair follicle stem cells (gHFSCs). (A, B) Gene expression and distribution in all treated gHFSCs samples. (C, D) Genes Pearson's correlation coefficient and FPKM correlation of all treated gHFSCs samples. (E) K-means cluster analysis of genes expression tendencies in all treated gHFSCs samples.
