# Supplementary material for: Integrative and comparative analysis of whole-transcriptome sequencing in circCOL1A1-knockdown and circCOL1A1-overexpressing goat hair follicle stem cells
Source: Anim Biosci. 2025 Feb 27;38(6):1116–39. doi: 10.5713/ab.24.0816 (PMC12061571; doi:10.5713/ab.24.0816)
Supplement: Supplementary file 10 [file ab-24-0816-Supplementary-10.pdf]

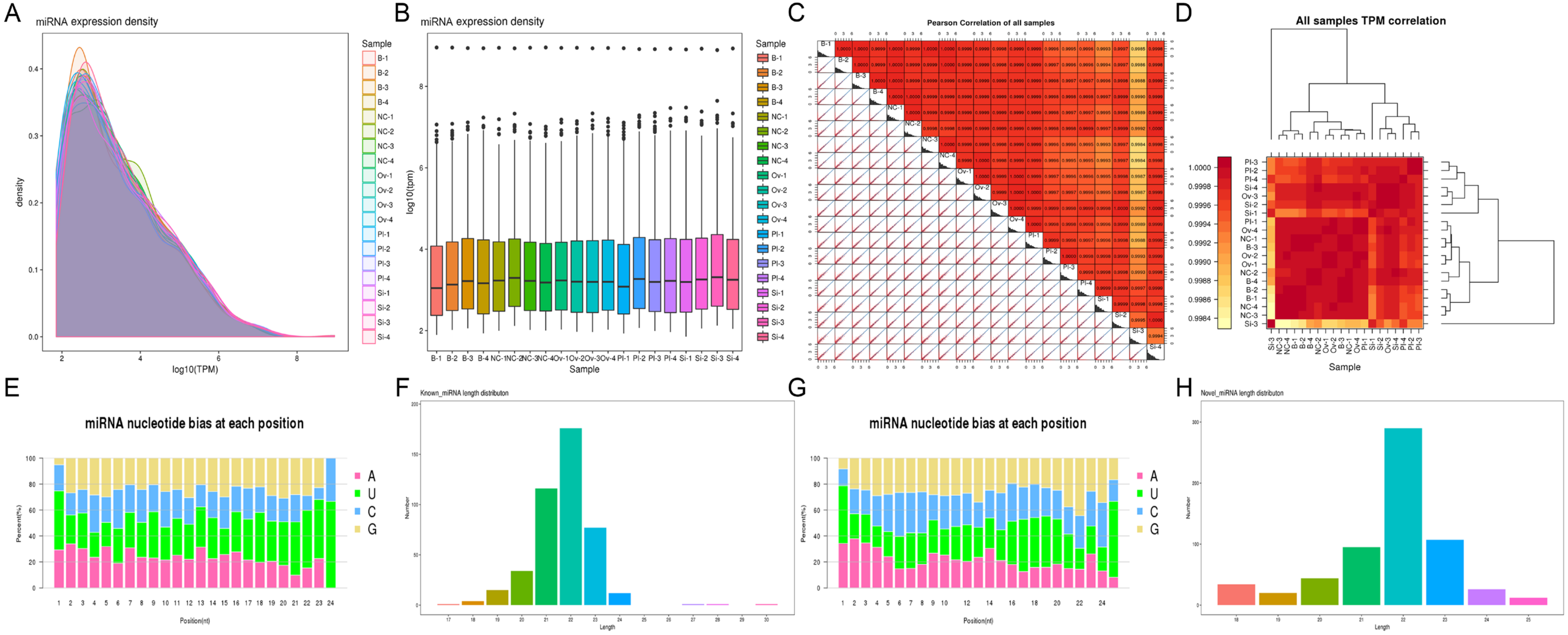

Supplement 10. Characteristics of miRNAs in circCOL1A1-overexpressing and circCOL1A1-knockdown goat hair follicle stem cells (gHFSCs). (A, B) miRNAs expression and distribution in all treated gHFSCs samples. (C, D) miRNAs Pearson’s correlation coefficient and TPM correlation of all treated gHFSCs samples. (E, F) Nucleotide bias of known miRNAs at each position and their length distribution. (G, H) Nucleotide bias of novel miRNAs at each position and their length distribution.
