# Supplementary material for: Integrative and comparative analysis of whole-transcriptome sequencing in circCOL1A1-knockdown and circCOL1A1-overexpressing goat hair follicle stem cells
Source: Anim Biosci. 2025 Feb 27;38(6):1116–39. doi: 10.5713/ab.24.0816 (PMC12061571; doi:10.5713/ab.24.0816)
Supplement: Supplementary file 11 [file ab-24-0816-Supplementary-11.pdf]

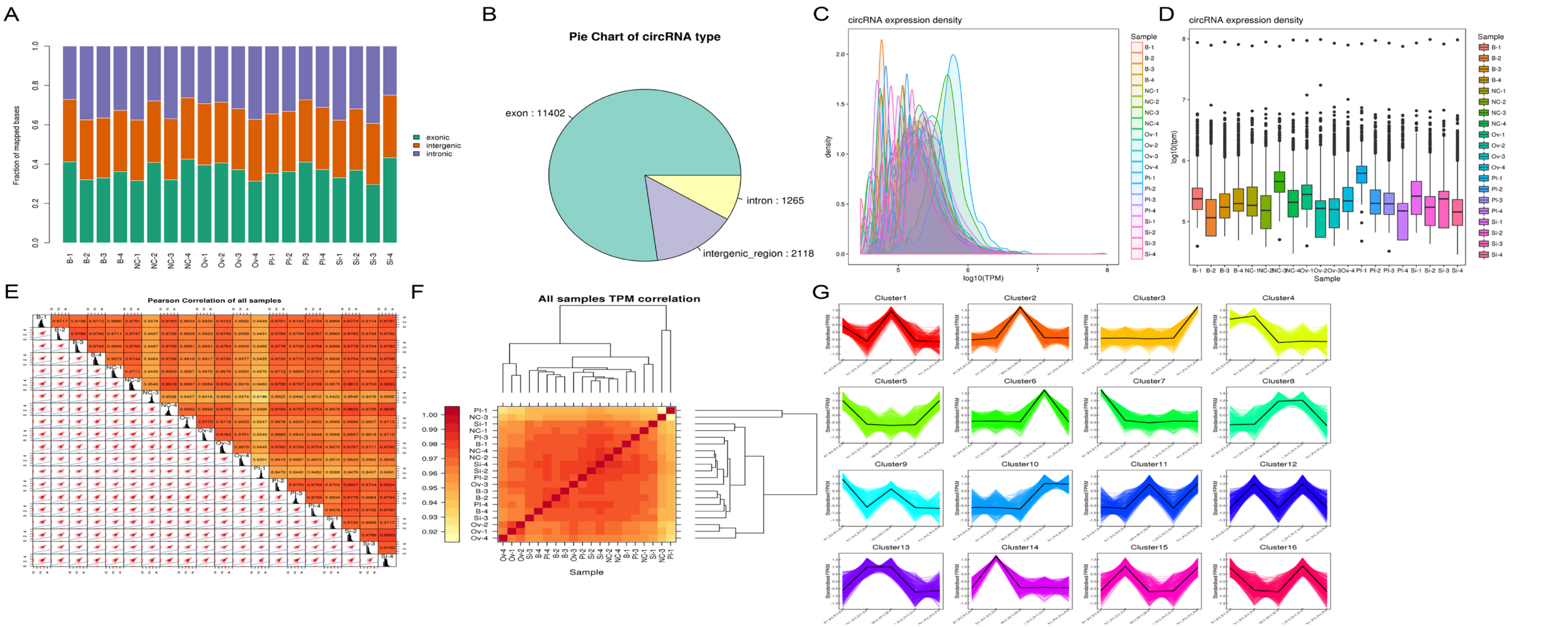

Supplement 11. Characteristics of circRNAs in circCOL1A1-overexpressing and circCOL1A1-knockdown goat hair follicle stem cells (gHFSCs). (A, B) circRNAs types and categories. (C, D) circRNAs expression and distribution in all treated gHFSCs samples. (E, F) Pearson's correlation coefficient and TPM correlation of circRNAs in all treated gHFSCs samples. (G) K-means cluster analysis of circRNAs expression tendencies in all treated gHFSCs samples.
